# Generation of Probabilistic Bits by Exploiting Orthogonal Spin Currents in Magnetic Trilayers

**DOI:** 10.1002/advs.202524371

**Published:** 2026-07-23

**Authors:** Donghyeon Han, Chaehyeon Shin, Seok‐Jong Kim, Yunho Jang, Daekyu Koh, Taehwan Kim, Jaeheon Jung, Geon‐Woo Baek, Minseok Kang, Eunseok Kim, Geun‐Hee Lee, Jeongchun Ryu, Makoto Kohda, Junsaku Nitta, Kab‐Jin Kim, Jongsun Park, Kyung‐Jin Lee, Byong‐Guk Park

**Affiliations:** ^1^ Department of Materials Science and Engineering KAIST Daejeon South Korea; ^2^ School of Electrical Engineering Korea University Seoul South Korea; ^3^ Department of Physics KAIST Daejeon South Korea; ^4^ Department of Materials Science Tohoku University Sendai Japan; ^5^ Center for Science and Innovation in Spintronics (Core Research Cluster) Tohoku University Sendai Japan; ^6^ Quantum Materials and Applications Research Center National Institute for Quantum Science and Technology Gunma Japan; ^7^ NTT Basic Research Laboratories Atsugi Japan

**Keywords:** magnetic trilayers, orthogonal spin currents, probabilistic bits, spin‐orbit torque

## Abstract

Probabilistic bits (p‐bits), non‐deterministic classical bits fluctuating between two digital states, constitute a core element in probabilistic computing. This adeptly addresses computationally complex problems, offering some of the envisioned capabilities of quantum computers while mitigating their major challenges such as shielding, cooling, and scalability. A recent study demonstrated the successful integer factorization using nanoscale magnetic tunnel junction (MTJ)‐based p‐bits. However, their stochasticity originates from the superparamagnetic properties of nanoscale MTJs, rendering p‐bits sensitive to the temperature and the dimensions of the device. Here, we demonstrate reliable p‐bits based on stochastic spin‐orbit torque (SOT) switching in micrometer‐sized magnetic trilayers. In a Fe/Ti/CoFeB structure, two spin currents with orthogonal spin polarizations are generated from the Fe/Ti bilayer, and their magnitude and sign determine SOT switching polarity of the top perpendicular CoFeB layer. This enables systematic control of the probability of having a ‘UP’ magnetization state of the CoFeB by either an external magnetic field or applied current for SOT switching. Furthermore, by harnessing the generated p‐bit streams, we effectively demonstrate invertible AND gate operations and stochastic neural networks with improved energy and area efficiency, thus highlighting the potential utility of our p‐bits in device applications.

## Graphical abstract

Fe/Ti/CoFeB trilayers generate orthogonal spin currents that drive stochastic spin–orbit‐torque switching for probabilistic‐bit operation. The switching probability is continuously controlled by the in‐plane magnetic field and drive current, enabling tunable random bit generation. Invertible AND logic and multiply–accumulate operations demonstrate the potential of this CMOS‐compatible architecture for scalable, energy‐efficient probabilistic computing.

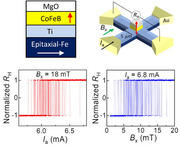

## 1 Introduction

With the emergence of big data and the growing demand for complex tasks, conventional computing technology based on binary bits is facing challenges in terms of increasing computational energy and time [1]. An alternative computing scheme is quantum computing that exploits quantum bits, which are represented by a superposition of two quantum states [2]. Owing to the quantum mechanical properties, quantum computers can utilize multiple states simultaneously, making it possible to efficiently solve difficult problems including integer factorization, optimization problems, and quantum simulations [3, 4, 5]. Despite significant advances in quantum computing technology, however, most quantum hardware operates at cryogenic temperatures, which entails significant energy and space consumption [6, 7]. Another unconventional computing technology is probabilistic computing, which utilizes probabilistic bits (p‐bits), non‐deterministic classical bits fluctuating between two digital states with tunable probability [8]. Because probabilistic computing shares similar concepts, it can replace quantum computing in some specific tasks: it efficiently accelerates randomized algorithms in various areas of artificial intelligence, such as stochastic neural networks [9] and Bayesian inference [10], as well as in complex computation problems, including energy‐based optimization problems [11] and quantum Monte Carlo algorithms [12]. Notably, p‐bits can be implemented in classical hardware, and therefore probabilistic computing offers the capabilities of room temperature operation and scalability.

The proficient generation of p‐bits is of great importance in achieving probabilistic computing. While conventional CMOS technology can generate p‐bits, it requires thousands of transistors, incurring substantial energy and area costs [13]. Therefore, various emerging devices with inherent stochasticity have been investigated for generating p‐bits [14, 15, 16, 17, 18], as they simplify integration into electronic hardware. The stochastic properties of these devices include magnetization switching in magnetic tunnel junctions (MTJs) [14, 15], threshold switching in diffusive memristors [16], voltage‐triggered metal‐insulator transition in vanadium oxide nanodevices [17], and electric‐field controlled resistance switching in perovskite nickelates doped with hydrogen [18]. In particular, p‐bits based on stochastic nano‐MTJs have successfully demonstrated integer factorization [13, 19] and traveling salesman problem [20], and in situ learning of weights and biases in a Boltzmann machine [21]. They utilize thermal fluctuations of magnetization direction between two states and enable the control of the probability of having a particular state with an external voltage. This technology offers advantageous features for p‐bit implementations due to its compatibility with standard CMOS processes, scalability, and high endurance, as demonstrated by the successful commercialization in embedded MRAM applications [22, 23, 24]. However, since the stochasticity originates from the superparamagnetic properties of the nanoscale MTJs [25, 26, 27], the p‐bits can be sensitive to temperature and device dimensions.

Here, we demonstrate the reliable generation of p‐bits based on stochastic spin‐orbit torque (SOT) switching in an epitaxial Fe‐based magnetic trilayer, where the probability of SOT switching is determined by the competition between two spin currents with orthogonal spin polarizations. In a micrometer‐sized Hall bar device with an Fe/Ti/CoFeB/MgO structure, where the bottom in‐plane magnetized Fe was epitaxially grown on a MgO substrate, the switching polarity of the top perpendicular magnetized CoFeB layer is systematically controlled using either an external magnetic field or input current. Notably, the probability of an ‘UP’ magnetization state demonstrates binary stochastic neuron behavior. Anomalous Hall loop shift experiments and micromagnetic simulations reveal that the stochastic SOT switching in the trilayer derives from the competition between the SOTs induced by two orthogonal spin currents carrying *y*‐ and *z*‐spin polarizations, respectively. Finally, we demonstrated the applicability of our p‐bits through circuit simulations of an invertible AND gate and stochastic neural network. In particular, we found that the energy and area consumption of the neural network employing our p‐bits for inference operation were significantly reduced compared to those using CMOS‐based random number generators. By combining the advantages of SOT and MRAM technologies, our approach holds promising prospects for low power consumption, high speed, and scalable probabilistic circuit implementation.

## 2 Results

### 2.1 Spin‐Orbit Torque Switching in Epitaxial‐Fe/Ti/CoFeB Trilayer

**FIGURE 1:**
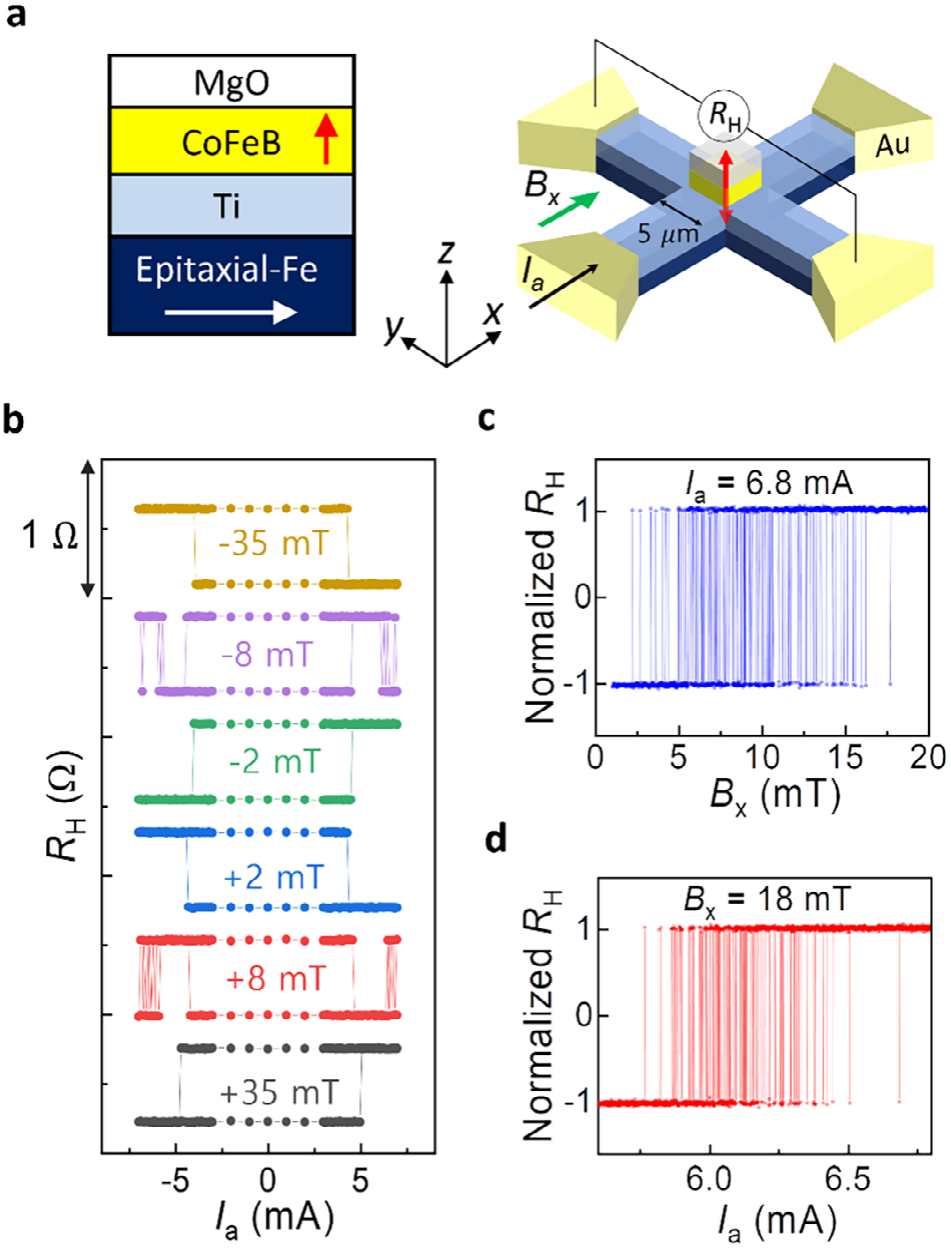
SOT switching measurement of an Fe/Ti/CoFeB trilayer. (a) Schematic illustration of the trilayer structure and measurement configuration. (b) Current‐induced SOT switching loops with different in‐plane magnetic fields *B_x_*. (c) Field (*B_x_*)‐dependent SOT switching curve with a constant *I*_a_ of + 6.8 mA. (d) Current (*I*_a_)‐dependent SOT switching curve with a constant *B_x_* of 18 mT.

To implement SOT‐based p‐bits, we employed an Fe (2 nm)/Ti (3 nm)/CoFeB (1 nm)/MgO (3 nm) trilayer. In this structure, the bottom Fe layer, epitaxially grown on an MgO(100) substrate, exhibits in‐plane biaxial magnetic anisotropy with easy axes along the [100] and [010] directions and hard axes along the [110] and [1$\bar{1}$0] directions, while the top CoFeB layer exhibits perpendicular magnetic anisotropy (Supporting Information S1). The Ti spacer layer was employed because it enables the development of perpendicular magnetic anisotropy in the top CoFeB layer while possessing a relatively small spin Hall angle. The latter minimizes the spin‐current contribution arising from the spin Hall effect in Ti, allowing the spin current generated at the Fe/Ti interface to dominate the SOT response. The samples were fabricated into a Hall bar device with a width of 5 µm, featuring a 4 µm‐sized CoFeB ferromagnetic island (Figure 1a).

We first conducted SOT switching experiments under different in‐plane magnetic fields (*B_x_*). Figure 1b shows the anomalous Hall resistance (*R*_H_) as a function of the applied current (*I*_a_) with various values of *B_x_*. Here, the pulse width of *I*_a_ is 100 µs. When *B_x_* is large (±35 mT), deterministic SOT switching is observed, with its switching polarity determined by the sign of *B_x_*: (counter‐)clockwise for a negative (positive) *B_x_*. This SOT switching polarity is consistent with that of a conventional Ta/CoFeB/MgO bilayer (Supporting Information S2). Notably, deterministic SOT switching is also observed for a small *B_x_* (±2 mT) in this structure, but with reversed switching polarity. This behavior is attributed to spin currents generated at the Fe/Ti interface. Unlike conventional heavy‐metal/ferromagnet bilayer structures, in which spin currents with in‐plane spin polarization along the *y* direction (σ_*y*_) are predominantly generated, magnetic trilayers can also generate spin currents with out‐of‐plane (σ_*z*_) polarization via spin‐orbit precession [28, 29]. Such spin currents can also arise from the magnetic spin Hall or spin‐swapping effects in magnetic trilayers [30, 31]. Regardless of the microscopic origin, the generation of σ_*z*_‐polarized spin currents requires an in‐plane Fe magnetization component along the current direction. Accordingly, all SOT measurements in this work were performed with *B_x_* applied parallel to the current direction along the Fe[100] easy axis. In contrast, when *B_x_* was applied along the hard axis, the Fe magnetization was rotated away from the current direction, and consequently no field‐free SOT switching behavior was observed (Supporting Information S3). Although the Fe magnetization direction is aligned by *B_x_* in the present study, a similar magnetization configuration could be achieved by employing materials with large in‐plane magnetic anisotropy [32] or introducing exchange bias via an additional antiferromagnetic layer [33].

Interestingly, for an intermediate *B_x_* (± 8 mT), the switching polarity changes depending on the magnitude of applied current, and the switching becomes stochastic at the transition field region. These behaviors for a small and intermediate *B_x_* were not observed in the bilayer sample (Supporting Information S2). We further examined the stochastic switching behavior as functions of *I*_a_ and *B_x_*. Figure 1c demonstrates the dependence of *R*_H_ on the magnitude of *B_x_* while applying a constant *I*_a_ =   + 6.8 mA. For a small (large) *B_x_*, the ‘UP’ (‘DOWN’) magnetization state is favored, consistent with the results shown in Figure 1b. Notably, the probability of the ‘UP’ magnetization state gradually increases with *B_x_*. A similar behavior is seen in Figure 1d, where the probability of the ‘UP’ magnetization changes with *I*_a_ under a constant *B_x_* = 18 mT. It is worth noting that these results can be represented by a binary stochastic neuron (BSN), mathematically described as *m_i_* =  *sgn*[*tanh* *I_i_* − *rand*(−1, 1)], where *I_i_* is the input and *m_i_* is the response to the input, which correspond to *B_x_* or *I*_a_ and the magnetization direction (or *R*_H_) in our trilayer sample, respectively.

Notably, stochastic switching behavior is not limited to epitaxial systems and is also observed in a polycrystalline CoFeB/Ta/CoFeB structure grown on a thermally oxidized Si substrate (Supporting Information S4). These characteristics–stochastic switching behavior in magnetic trilayers and its controllability–form the basis for the p‐bit implementation discussed in the later chapters.

### 2.2 Control of the SOT Switching Probability

**FIGURE 2:**
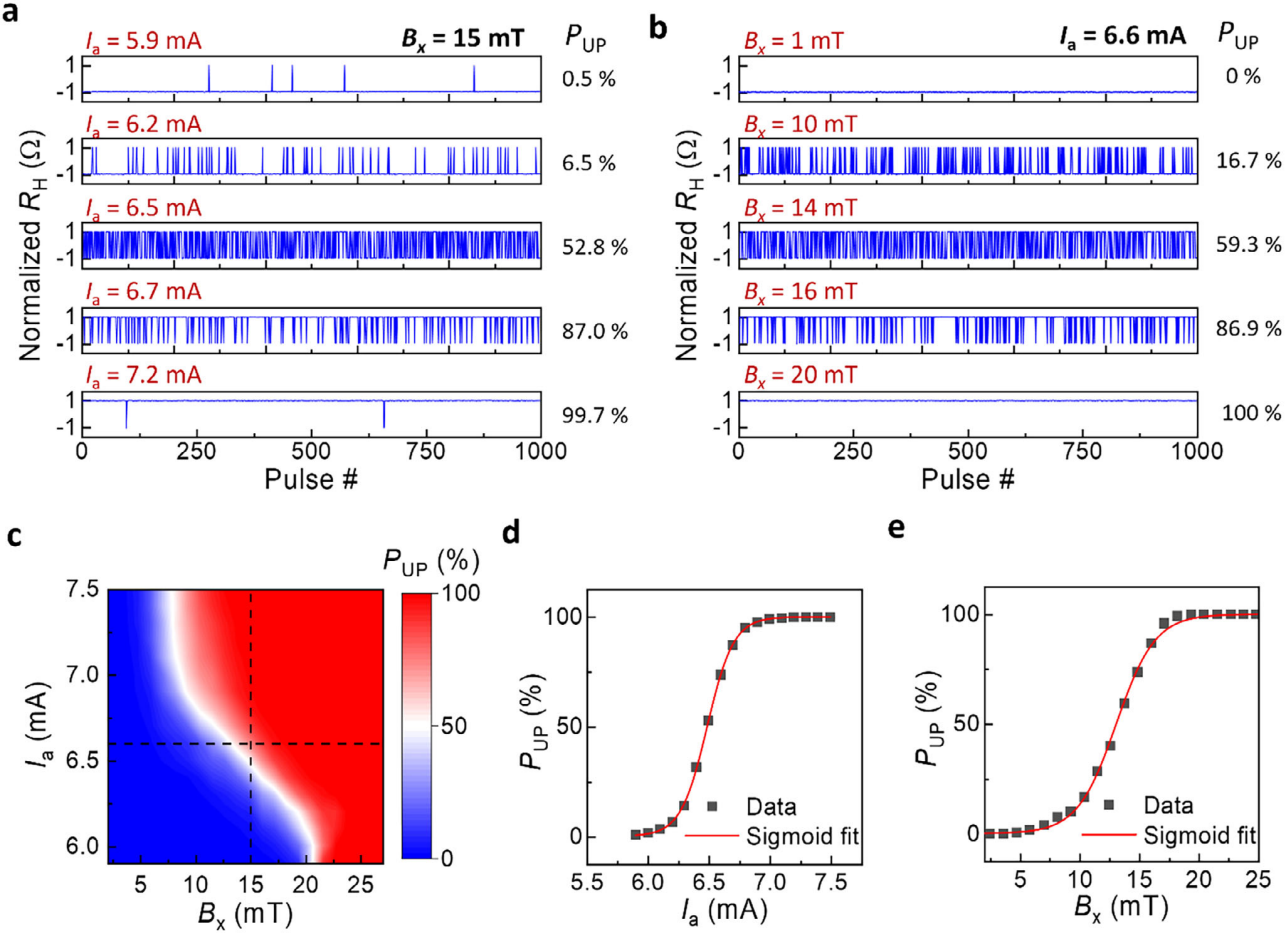
Control of probabilistic SOT switching. (a, b) Repetitive SOT switching for 1000 trials for different *I*_a_ with a constant *B_x_* of 15 mT (a) and different *B_x_* with constant *I*_a_ of 6.6 mA (b). (c) *P*_UP_ as functions of *B_x_* and *I*_a_, where the blue and red colors represent *P*_UP_ = 0 and *P*_UP_ = 100, respectively, and the white area indicates the transition regions of *P*_UP_. (d, e) Line profiles of *P*_UP_ obtained from vertical (d) and lateral (e) dashed lines in Figure 2c. The red curves indicate the sigmoid fittings.

We next demonstrate the systematic control of stochastic switching using two parameters, *I*_a_ and *B_x_*, which are essential for implementation of p‐bits. For this measurement, we applied a pulse current *I*_a_ and subsequently monitored the magnetization direction by measuring *R*_H_ for each cycle (Methods). For a total of 1000 switching trials, we counted the number of ‘UP’ states and evaluated the probability of having an ‘UP’ state (*P*_UP_). Figure 2a shows the results, where *I*_a_ is varied from 5.9 mA to  7.2 mA while maintaining a constant *B_x_* of 15 mT. Note that the raw *R*_H_ data are presented in Supporting Information S5, and the normalized *R*_H_ values of ±1 represent the fully ‘UP’ and ‘DOWN’ magnetization states. For *I*_a_ = 5.9 mA, the SOT switching predominantly results in the magnetization direction being ‘DOWN’, *P*_UP_ = 0.5%. With increasing *I*_a_, the switching becomes stochastic and *P*_UP_ gradually increases. At *I*_a_ = 6.5 mA, *P*_UP_ becomes 52.8%, corresponding to nearly equal probabilities of having ‘UP’ and ‘DOWN’ states. *P*_UP_ continues to increase and reaches 99.7% for *I*_a_ = 7.2 mA. This demonstrates that *P*_UP_ is effectively controlled by *I*_a_. Figure 2b shows the modulation of the switching probability *P*_UP_ using *B_x_*, with a constant *I*_a_ of 6.6 mA. Similarly, *P*_UP_ gradually increases from 0% to 100% with increasing *B_x_*. Notably, while we demonstrated probabilistic switching using a 100‐µs current pulse, it can also be observed on the nanosecond timescale (Supporting Information S6).

Figure 2c presents a summary of *P*_UP_ as functions of *I*_a_ and *B_x_*. The blue and red colors indicate *P*_UP_ = 0 and *P*_UP_ = 100, respectively, representing deterministic switching. The transition regions of *P*_UP_ show stochastic switching behavior. Figure 2de shows a line profile of *P*_UP_, indicated as the vertical (lateral) dashed line in Figure 2c, demonstrating that *P*_UP_ can be gradually controlled from 0% to 100% by *I*_a_ (*B_x_*). The red fitting curves show that *P*_UP_ is well described by a sigmoid function. Additionally, we evaluated the statistical properties of our p‐bit by examining the autocorrelation function and performing the NIST statistical tests (Supporting Information S7). These results demonstrate that our p‐bit device generates statistically random and uncorrelated bit streams.

Our p‐bits exhibit several distinctive features. First, the switching probability (*P*_UP_) does not depend on the initial magnetization state, allowing p‐bit generation without an explicit initialization process (Supporting Information S8). This initialization‐free operation is advantageous compared with existing switching‐based probabilistic devices [34, 35, 36], in which stochastic behavior is typically realized through probabilistic switching from a predefined initial state and therefore requires reset pulses and additional circuit elements. By eliminating repeated initialization, our approach simplifies circuit design and reduces power consumption. Second, SOT‐based probabilistic switching in a micron‐sized sample occurs between two distinct binary states, corresponding to up and down magnetization directions without any intermediate states (Supporting Information S5 and S9). This is consistent with the role of the σ_*z*_‐ polarized spin current in assisting in domain‐wall depinning, thereby suppressing intermediate multidomain states (see further discussion in Supporting Information S10). Third, since p‐bit generation in our device is based on the competition between the orthogonal SOTs (to be discussed later), the stochastic switching does not require lowering the energy barrier to the thermal energy scale (∼*k*_B_T), making it less sensitive to external factors such as temperature or variations in device dimensions. Finally, for a projected nanoscale implementation of the proposed p‐bit, benchmarking against representative CMOS‐based random number generators implemented in a 28‐nm technology node [37, 38] indicates significant reductions in both energy consumption and cell area (see details in Supporting Information S11). Furthermore, by leveraging experimentally demonstrated sub‐nanosecond SOT switching in structurally similar trilayer devices [39], the estimated bit‐generation energy can be further reduced to a level comparable to that of stochastic MTJs (Supporting Information S12). We note that an external magnetic field (*B_x_*) was employed for p‐bit operation in the present study; however, field‐free implementation should be feasible by incorporating an internal in‐plane bias field, as demonstrated in established field‐free SOT‐switching schemes [28, 40, 41, 42]. Taken together, these results highlight the advantages of p‐bit generation based on the controlled competition between orthogonal SOTs at the device‐physics level.

### 2.3 Quantification of Orthogonal Spin Currents in a Magnetic Trilayer

To understand stochastic switching behavior of our sample, we analyzed the SOTs generated in the magnetic trilayer through the AHE loop shift measurements [43]. The change in the switching polarity with the external magnetic field suggests the presence of two competing SOT contributions with opposite signs, with their relative magnitudes varying with the applied magnetic field. One contribution arises from SOT generated by σ_*y*_‐polarized spin currents, which have the same symmetry as the spin Hall and/or Rashba‐Edelstein effects [44, 45, 46]. This σ_*y*_‐induced SOT produces an effective out‐of‐plane field that leads to an AHE loop shift in the presence of the in‐plane magnetic field *B_x_*. The other contribution originates from the SOT generated by σ_*z*_‐polarized spin currents, which also results in an AHE loop shift. In contrast to the σ_*y*_ contribution, the direction of shift depends on the magnetization direction of the bottom magnetic layer and is independent of *B_x_*.

**FIGURE 3:**
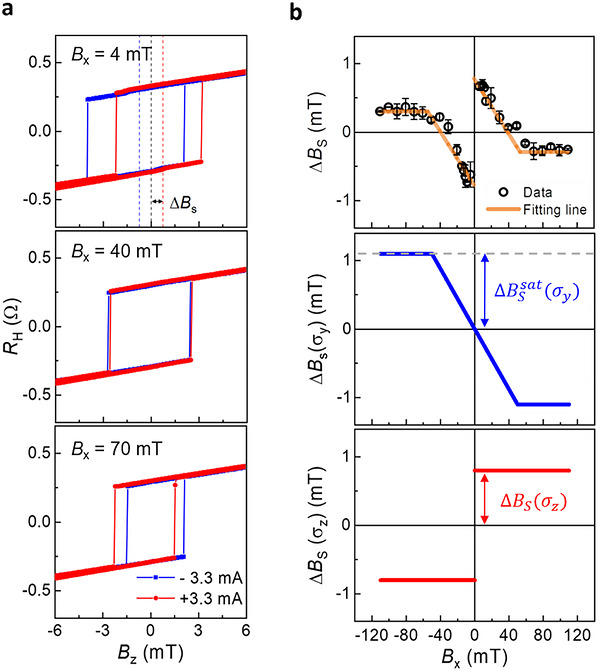
Quantification of SOTs using anomalous Hall effect (AHE) loop shift. (a) AHE hysteresis loops measured under d.c. currents of ± 3.3 mA at *B_x_* of 4, 40, and 70 mT. (b) Decomposition of the measured AHE loop shift Δ*B*_S_ into field‐dependent and field‐independent components. Top panel: measured Δ*B*_S_ versus *B_x_* (open circle) and a fitting line. Middle panel: field‐dependent component Δ*B*_S_(σ_*y*_), attributed to the σ_*y*_‐polarized spin current. The saturation value $\;\Delta B_{S}^{sat}\left(\sigma_{y}\right)$, used to extract $\theta_{SH}^{eff}\left(\sigma_{y}\right)$, is indicated by the arrow. Bottom panel: field‐independent Δ*B*_S_(σ_*z*_), attributed to the σ_*z*_‐polarized spin current. The field‐independent offset Δ*B_S_* (σ_*z*_), used to extract $\theta_{SH}^{eff}\left(\sigma_{z}\right)$, is indicated by the arrows.

Figure 3a shows the AHE measurement results with d.c. currents *I*_d.c._ = ±3.3 mA under different *B_x_*. It was found that the AHE loop shifts to the right (left) for positive (negative) current for small *B_x_* (4 mT), while the shift direction is reversed for large *B_x_* (70 mT). There is no loop shift around *B_x_* = 40 mT. Figure 3b shows the loop shift Δ*B*_S_ as a function of *B_x_*, where $\Delta B_{S}=\left[B_{S}\left(I_{d.c.}^{+}\right)-B_{S}\left(I_{d.c.}^{-}\right)\right]\;/2$, $B_{S}=\left[B_{S}^{+}+B_{S}^{-}\right]/2$, $B_{S}^{\pm }$ is the positive or negative coercive field, and $I_{d.c.}^{\pm }$ is positive or negative *I*_d.c._. Here, each data point of Δ*B*_S_ represents average values obtained from repeated measurements, and the error bars indicate the corresponding standard deviations (Supporting Information S13). To quantitatively evaluate the effective SOT fields caused by the two spin‐current components, we separated the measured Δ*B*_S_ into field‐dependent and field‐independent components based on their distinct *B_x_* dependences, which are plotted in the middle and bottom panels of Figure 3b, respectively. The field‐dependent component, Δ*B*_S_(σ_*y*_), exhibits a linear increase with *B_x_* at small fields and saturates above *B_x_* = 54 mT. This behavior is consistent with that of a conventional Ta/CoFeB/MgO structure, where only σ_*y*_ is generated (Supporting Information S2). In contrast, the field‐independent component, Δ*B*_S_(σ_*z*_), appears as a finite offset in Δ*B*_S_, whose polarity reverses upon reversal of *B_x_*. The polarity reversal of Δ*B*_S_(σ_*z*_) originates from the reversal of the bottom Fe magnetization induced by the change in the sign of *B_x_*. This result explains the switching behavior shown in Figure 1. For a large (small) *B_x_*, the SOT switching is dominated by the spin current with σ_*y*_ (σ_*z*_), and thus the switching polarity depends on the magnitude of *B_x_*. For an intermediate *B_x_*, the SOTs with opposite polarity are similar in magnitude, resulting in stochastic switching whose probability is controlled by *B_x_*. This will be discussed in more detail later with micromagnetic simulations. Furthermore, we extracted effective spin Hall angles $\theta_{SH}^{eff}$ of each spin current using $\theta_{SH}^{eff}=\frac{2eM_{s}t_{F}}{\hbar }\times \chi_{SOT}$, where *M_s_* and *t_F_* are the saturation magnetization and thickness of the top CoFeB layer, *e* is the fundamental charge, ℏ is the reduced Planck constant. The χ_*SOT*_ values for σ_*y*_ and σ_*z*_ contributions were determined from the saturation value $\Delta B_{S}^{sat}\left(\sigma_{y}\right)$ and the field‐independent offset Δ*B_S_* (σ_*z*_), respectively, divided by *J*_d.c._(Supporting Information S14). We obtained effective spin Hall angles, $\theta_{SH}^{eff}\left(\sigma_{y}\right)=-$0.024 ± 0.0012 and $\theta_{SH}^{eff}\left(\sigma_{z}\right)=$0.018 ± 0.0018, where the error bars represent the uncertainty associated with the linear fitting procedure. While the effective spin Hall angles in the present trilayer are sufficient to demonstrate the proposed p‐bit concept, further enhancement will be beneficial for low‐power applications. Such improvements may be achieved through materials and interface engineering, including optimization of the ferromagnet/non‐magnet heterostructure and the exploration of alternative trilayer systems with higher spin‐current efficiencies.

### 2.4 Micromagnetic Analysis of SOT Switching in a Magnetic Trilayer

In this section, we discuss probabilistic switching in magnetic trilayers by numerical and analytical investigations based on the Landau‐Lifshitz‐Gilbert (LLG) equation. For the top ferromagnet (FM) of the magnetic trilayer system, the LLG equation, including damping‐like SOT (DLT), is given by

(1) $$\begin{array}{c} \partial_{t}\;\hat{m}=-\gamma \hat{m}\times \left(B_{eff}+B_{th}\right)+\alpha \hat{m}\times \partial_{t}\hat{m}+\gamma h_{D}\hat{m}\times \left(\hat{m}\times \hat{\sigma }\right) \end{array}$$

where terms on the right‐hand side describe torques from precession, damping, and DLT, respectively. Here $\hat{m}$ is the unit vector of top FM magnetization, γ is the gyromagnetic ratio, and $B_{eff}\left(=B_{k}\left(\hat{m}\;\cdot \hat{z}\right)\hat{z}+B_{x}\hat{x}\;\right)$ is the effective field including magnetic anisotropy (*B_k_*) and an external field along the *x*‐direction (*B_x_*). **B**_*th*_ is the thermal fluctuation field, which follows 〈**B**_*th*_(*t*)〉 =  0 and $\left\langle B_{th}\left(t\right)B_{th}\left(t^{\prime }\right)\right\rangle =\frac{2k_{B}T\alpha }{\gamma VM_{s}}\;\delta \left(t-t^{\prime }\right)$ [47], where *k_B_* is the Boltzmann constant, *T* is the temperature, *V* is the volume of the top FM, *M_S_* is the saturation magnetization, and α is the damping constant. *h_D_* =  ℏθ_*SH*, *D*_ *J*/2*eM_s_t_F_*, where θ_*SH*,*D*_ is the effective spin Hall angle of the DLT, *J* is the current density, *t_F_* is the thickness of the top FM, and $\hat{\sigma }$ is a spin polarization vector defined as $\hat{\sigma }=\left(0,\cos\eta ,\sin\eta \right)$. The parameter η determines the ratio between σ_*y*_ and σ_*z*_, i.e., pure σ_*y*_ for η  = 0° or 180° and pure σ_*z*_ for η  = 90° or 270°. Here, we assumed the existence of DLT from *z*‐spin, even though we only measured the field‐like torque caused by *z*‐spin in Figure 3, since the magnetization switching induced by *z*‐spin DLT has already been confirmed theoretically and experimentally in magnetic trilayer systems [32, 48, 49]. There are two mechanisms for current‐induced magnetization switching in our trilayer sample. One is due to the *z*‐spin (*z*‐SOT), which is the same as the spin‐transfer torque (STT) mechanism [50, 51]. The other is due to the *y*‐spin (*y*‐SOT), which is conventional SOT [52]. Switching current densities induced by *z*‐SOT and *y*‐SOT can be derived from Equation (1) [53, 54, 55] (See Supporting Information S15 for details of equation derivations),

(2) $$\left|J_{z-SOT}\right|=\pm \alpha \frac{2e}{\hbar }\frac{M_{s}t_{F}}{\theta_{SH,D}}B_{k}\left(1+\frac{B_{x}^{2}}{B_{K}^{2}}\right)\frac{1}{\sin\eta }$$

(3) $$\left|J_{y-SOT}\right|=\pm \frac{2e}{\hbar }\frac{M_{s}t_{F}}{\theta_{SH,D}}\left(\frac{B_{k}}{2}\pm \frac{\left|B_{x}\right|}{\sqrt{2}}\right)\frac{1}{\cos\eta }$$

To demonstrate probabilistic switching in the trilayer, two conditions must be satisfied. First, switching polarities favored for *z*‐SOT and *y*‐SOT should be opposite. For example, if *z*‐SOT favors ‘DOWN’ magnetization, *y*‐SOT should favor ‘UP’ magnetization in the presence of *B_x_*. According to the LLG equation, opposite switching polarities occur when σ_*y*_ and σ_*z*_ have opposite signs (90° < η < 180° or 270° < η < 360°). Second, *B_x_* or *J* should fall within a range where *z*‐SOT and *y*‐SOT are comparable in magnitude to induce stochastic switching. We derived the condition that the switching probability is 50% (*P*_UP_ = 50%) as follows:

(4) $$J_{50\%}=\pm \frac{2e}{\hbar }\frac{M_{s}t_{F}}{\theta_{SH,D}}\frac{B_{x}}{\sin\eta }$$

**FIGURE 4:**
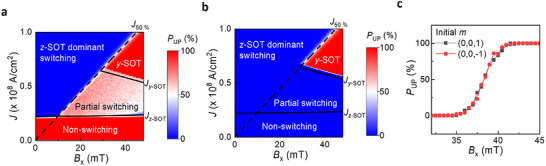
LLG simulations of magnetization switching in magnetic trilayer. (a, b) *P*_UP_ of the magnetic trilayer as functions of *B_x_* and *J* for initial magnetization of ‘UP’ (a) and ‘DOWN’ (b) directions. Blue (red) color indicates *P*_UP_ = 0% (*P*_UP_ = 100%). The black solid lines represent switching current densities of *z*‐SOT and *y*‐SOT obtained from Equations (2) and (3), respectively, and the black dotted line represents *P*_UP_ = 50% obtained from Equation (4). (c) Line profiles of *P*_UP_ versus *B_x_* obtained from the line scans in Figure 4a,b for a *J* of 0.85x10^8^ A/cm^2^. Parameters used for the simulations are as follows: *B_k_* =  0.2 T, *M_S_* =  500 kA/m, *T*  =  300 K, *V*  =  500 × 500 × 1 nm^3^, α  =  0.05, θ_*SH*,*D*_ =  0.2, *t_F_* =  1 nm, η  =  160°, current pulse width = 200 ns. Two‐hundred iterations were performed for calculation of the switching probability for each parameter set.

Note that by tuning *B_x_* or *J* near the conditions of Equation (4), *P*_UP_ can be modulated from 0% to 100%. Figure 4 shows *P*_UP_ as functions of *B_x_* and *J*. Here, we assumed η  =  160° so that the *z*‐SOT switching prefers ‘DOWN’ magnetization while the *y*‐SOT switching prefers ‘UP’ magnetization when *J* > 0 and *B_x_* > 0. Figure 4a shows the simulation results for the initial state of ‘UP’ magnetization, demonstrating four regions of different switching characteristics, where the black solid lines represent Equations (2) and (3) and the black dotted line represents Equation (4). Among the four regions, we are interested in the transition between the *z*‐SOT‐ and *y*‐SOT‐dominant switching regions. The *z*‐SOT‐ and *y*‐SOT‐dominant switching regions show deterministic switching. Near the boundary, the two mechanisms compete with each other, enabling probabilistic switching. The switching probability can be tuned by sweeping *J* or *B_x_* cross the boundary. Similar probabilistic switching is also observed for the initial state of ‘DOWN’ magnetization shown in Figure 4b. Note that in the non‐switching region, *J* is lower than *J*_*z* − *SOT*_ and *J*_*y* − *SOT*_, and in the partial switching region, *J* ≥ *J*_*z* − *SOT*_, but due to a large *B_x_*, switching is initial‐state dependent; this, however, is not focused upon in this study. Furthermore, we examined the stochastic switching regions in all other *J* or *B_x_* conditions (Supporting Information S16). Figure 4c shows *P*_UP_ as a function of *B_x_*, obtained from the line scan in Figure 4a,b, demonstrating identical *P*_UP_ profiles independent of the initial states, consistent with the results shown in Figure 2.

Note that the boundary slope between the *z*‐SOT and *y*‐SOT regions in the modeling (Figure 4a,b) differs from that observed in the experiment (Figure 2c). This discrepancy may be attributed to modifications of the magnetic anisotropy of CoFeB resulting from current‐induced Joule heating during the switching experiments, although further investigation is necessary to ascertain the underlying causes.

### 2.5 Invertible Logic and Probabilistic Neural Networks

**FIGURE 5:**
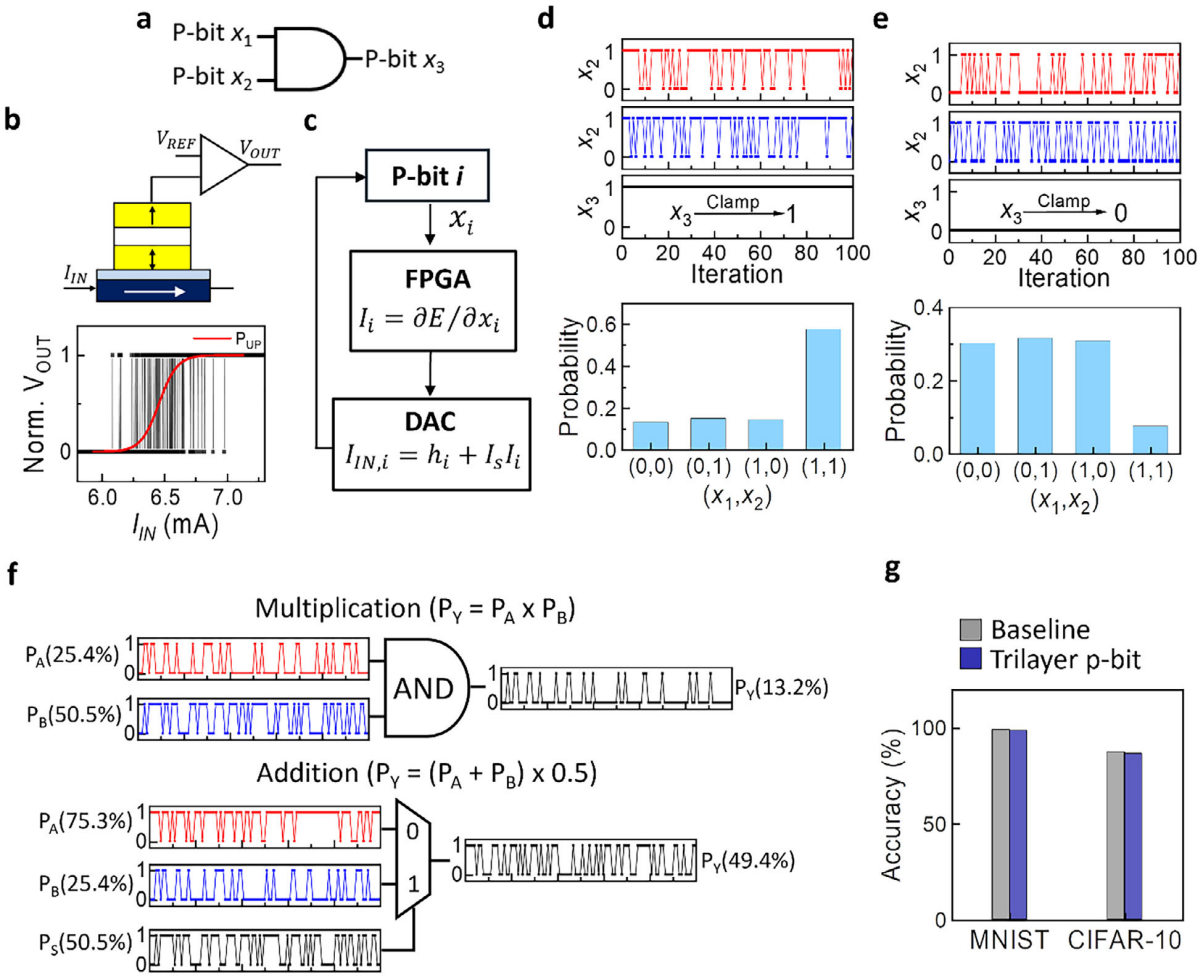
P‐bit‐based invertible logic and probabilistic neural network. (a) Illustration of an ‘AND’ gate with three p‐bit devices, where *x*_1_ and *x*_2_ are the inputs and *x*_3_ is the output. (b) Schematic of a trilayer‐based magnetic tunnel junction with p‐bit characteristics. The red curve represents *P*_UP_ obtained from the sigmoid function in Figure 2d, and *V_OUT_* is the corresponding output voltage representing a p‐bit of ‘0’ or ‘1’. (c) Schematic diagram of the p‐bit based ‘AND’ gate operation. The field programable gate array (FPGA) is programmed to outputs digital bits *I_i_* depending on inputs and the cost function. The digital to analog converter (DAC) updates new analog inputs using the relation. *I*_*IN*,*i*_ =  *h_i_* + *I_s_I_i_*. (d, e) The results of the ‘AND’ gate operation. Top panel: p‐bit streams of three p‐bit devices (*x*_1_, *x*_2_, *x*_3_) for 100 iterations. Bottom panel: the probabilities of inputs (*x*_1_, *x*_2_) obtained when output (*x*_3_) is set to ‘1’ (d) and ‘0’ (e). (f) MAC operations using the generated p‐bit streams, (top) ‘AND’ gate and (bottom) ‘MUX’ circuit operations. (g) Inference accuracy of p‐bit based neural network for MNIST and CIFAR‐10 datasets.

Finally, we demonstrate the advantages of our p‐bits by implementing them into two probabilistic computing applications using device simulations. The first is an invertible logic, which can predict inputs from a given output and vice versa. This is one of the difficult calculations for conventional digital computing because the answer may not be unique [56]. To this end, we constructed an ‘AND’ gate with three p‐bit devices, as illustrated in Figure 5a. Two of them correspond to inputs *x*_1_, *x*_2_ and the last represents output *x*_3_. We assumed that each p‐bit consists of a magnetic tunnel junction, where the probability of having an ‘UP’ magnetization state of a free layer and the resultant tunnel magnetoresistance is effectively controlled by the in‐plane current *I_IN_* (Figure 5b). Each p‐bit generates *x_i_* based on the sigmoid fitting curve in Figure 2c, with random noise included (see details in the Method). Figure 5c describes the ‘AND’ gate operation procedure based on p‐bits. For an ‘AND’ function satisfying *x*_1_ *x*_2_ = *x*_3_ , the cost function is defined as *E*  = (*x*_3_ − *x*_1_ *x*_2_)^2^ . Each p‐bit is driven by a synaptic input *I_i_*, which depends on the outputs of other p‐bits, obtained from the relation $I_{i}=\frac{\partial E}{\partial x_{i}}$. The calculated *I_i_* value is updated as a new input fed back into the p‐bits using a digital to analog converter (DAC) with the relation *I*_*IN*,*i*_ = *h_i_*  + *I_s_I_i_*, where *h_i_* is the external bias and *I_s_* is the correlation strength between the p‐bit devices. Here, we set *I_s_* = 0.2 mA, which results in *P*_UP_ = 80 (20) % when *I_i_* is 1 (‐1), and adjust *h_i_* to clamp the probability. Figure 5d,e shows the results of the reverse ‘AND’ operation with output values of “1” and “0”, respectively. First, we set output *x*_3_ to 1 using the external bias *h*_3_ while updating *x*_1_ and *x*_2_ with each iteration. From 1000 iterations, we obtained the probability P (*x*_1_, *x*_2_), as shown in the bottom panel in Figure 5d. For a given output *x*_3_ = 1, P (1, 1) is the highest, about 0.6, while P(0,0), P(1,0), and P(1,0) are less than 0.1. Similarly, for a given output *x*_3_ =  0 (Figure 5e), P (0,0), P(0,1), and P(1,0) show equal probabilities of about 0.3, while P(1,1) has the lowest probability of about 0.1. This demonstrates that the reverse ‘AND’ logic operations have been successfully achieved using our p‐bits devices. We also performed forward ‘AND’ gate logic operations using our p‐bits devices (Supporting Information S17). Note that other complex logic operations, such as full‐adder and factorization, can be realized by our p‐bits devices with a proper cost function.

The second application of the p‐bits is a probabilistic neural network [57, 58], where the multiplication and accumulation (MAC) operations are performed by the p‐bits. To conduct the MAC operation, we first generated p‐bit streams of 1000 bits with 16 different probabilities of forming 4 bits (Supporting Information S18). Figure 5f shows the results of the ‘AND’ gate and ‘MUX’ circuit operations, where only the calculation using 100 bits is shown here, while the full calculation with 1000 bits is shown in Supporting Information S19. For the ‘AND’ gate, two inputs of P_A_ = 25.4% and P_B_ = 50.5% give an output with a probability of 13.2%, which is approximately multiplication of the probabilities of the two inputs. For the ‘MUX’ circuit, two inputs of P_A_ = 75.3% and P_B_ = 25.4% with a selector (P_S_ = 50.5%) give an output with a probability of 49.4%, which is about half the sum of the probabilities of the two inputs. Leveraging p‐bits simplifies MAC operations, which reduces circuit costs. It is estimated that using 4‐bit p‐bits can decrease the product of energy and area (EAP) by 39% compared to CMOS‐based MAC operations, and an even greater reduction in EAP is expected by increasing the bit size (Supporting Information S20).

Based on this p‐bit‐based MAC architecture, we constructed neural networks with p‐bit‐based MAC operations and performed inference tests using the MNIST and CIFAR‐10 datasets (see Method for details). Figure 5g shows inference accuracy of 98.72% and 86.67% for the MNIST and CIFAR‐10 datasets, respectively, which are less than 1% of the 4‐bit baseline, demonstrating that the p‐bit‐based approach maintains competitive inference accuracy. In addition, we demonstrate that our p‐bits can be applied to two further computing tasks: integer factorization and the Max‐Cut problem, a representative NP‐hard optimization benchmark. Detailed methods and results are provided in Supporting Information S21. The successful demonstration of these computing tasks based on our p‐bits highlights their potential for probabilistic circuit implementation. Nevertheless, the practical realization of p‐bit computing will require the development of MTJ‐based, CMOS‐compatible hardware architectures beyond the present proof‐of‐concept simulation framework.

## 3 Conclusions

We demonstrate a novel approach to generate reliable p‐bits using stochastic SOT switching in a micrometer‐sized magnetic trilayer of an Fe/Ti/CoFeB/MgO structure. Using either an external magnetic field or input current, we can systematically manipulate the probability of having an ‘UP’ magnetization state, akin to binary stochastic neuron behavior. Anomalous Hall loop shift measurements and micromagnetic simulation results reveal that the stochastic switching is due to the competition between the SOTs induced by two orthogonal spin currents carrying *y*‐ and *z*‐spin polarizations, respectively. Furthermore, we showcase the practical utility of our p‐bits through circuit simulations, demonstrating invertible ‘AND’ gate operations and stochastic neural networks. Notably, these applications exhibit improved energy and area efficiency compared to conventional CMOS‐based approaches, paving the way for low‐power, high‐speed, and scalable probabilistic circuit implementations.

## 4 Methods

### 4.1 Sample Preparation

Trilayer structures comprising Fe/Ti/CoFeB/MgO/Ti layers were deposited on MgO (100) single‐crystalline substrates using magnetron sputtering with a base pressure of 8 × 10^−7^ Pa. During the Fe deposition, the substrate was heated to 200°C to promote epitaxial growth, while the other layers were deposited at room temperature.

### 4.2 Device Fabrication

The magnetic trilayers were patterned into Hall bar devices with a width of 5 µm and a length of 15 µm using photolithography and Ar ion milling. A 4‐µm‐diameter ferromagnetic island of CoFeB was then defined at the center of the Hall cross. Finally, contact pads of Ti (5 nm)/Au (100 nm) layers were deposited by sputtering followed by a lift‐off process.

### 4.3 Electrical Measurements

Current‐induced switching measurements were conducted by applying a pulsed current (*I*_a_) with a width of 100 µs and monitoring the magnetization direction of the top ferromagnet via anomalous Hall voltage using a 1 mA reading current after a delay of 83 ms. Repetitive switching experiments were carried out by iterating the above procedure without any initialization procedure.

### 4.4 Invertible AND Gate Operation

For the p‐bit based ‘AND’ gate operations, an energy‐based network was modeled to minimize the total energy through iterative updates. To achieve this, a Boltzmann machine was utilized, where the energy function is expressed as $E=-I_{0}\left(\sum_{i,j}J_{ij}x_{i}x_{j}+\sum_{i}h_{i}x_{i}\right)$, where *x_i_* =  sgn{rand(−1, 1) + tanh (*I_i_*)}, *I*_0_, *J_ij_*, and *h_i_* are the *i* ^th^ p‐bit output, the scaling factor, the weight of connecting two p‐bits, and the external bias, respectively. The p‐bits were iteratively updated to minimize the system's energy by interconnecting them according to $I_{i}=I_{0}\;\left(h_{i}+\sum_{i,j}J_{ij}x_{j}\right)$. In this configuration, output *x_i_* were produced by the p‐bit devices based on the sigmoid function shown in Figure 2d, where we introduced random noise to reflect the device's characteristics and performance. Specifically, we derived the magnitude of this random noise based on the mean and standard deviation values of the differences between the experimental data and the fitted sigmoid curve. This allowed us to introduce fluctuations in the switching probability that mirror the inherent stochastic behavior of the physical device. The FPGA plays a role in hardware implementation of the p‐computing network by interconnecting the p‐bit circuits and synaptic hardware to perform logic operations in real time, and the DAC generate the switching current *I*_*IN*,*i*_ according to the relation *I*_*IN*,*i*_ =   *h_i_* + *I_s_I_i_*. In this simulation, we functionally simulated the roles of these components within our Python environment to accurately replicate their behavior within the computational system.

### 4.5 Stochastic Neural Network

The inference test of CNNs is performed to evaluate the accuracy and effectiveness of trilayer p‐bit by comparing the results of p‐bit MAC operation against deterministic computations. Using experimentally generated 16 different p‐bit streams, a total of 100 streams were generated by measuring a 100‐bit p‐bit stream 10 times for each of 16 possible 4‐bit values. By randomly sampling p‐bit streams as MAC inputs one million times, p‐bit MAC operations were executed, and the results were compared with those from deterministic computing to calculate errors. Simulations with various inputs revealed a widening error range as the number of inputs increased. This approach enabled the quantitative evaluation in p‐computing implementation using our trilayer p‐bits, which were then integrated into a CNN for a comprehensive evaluation of inference accuracy. For MNIST [59] and CIFAR‐10 [60] inferences, the LeNet5 [61] and ResNet‐20 [62] networks were employed, respectively. Both networks were trained using floating‐point computations, while p‐computing was employed for inference with 4‐bit precision.

## Conflicts of Interest

The authors declare no conflicts of interest.

## Supporting information




**Supporting File**: advs76759‐sup‐0001‐SuppMat.pdf.

## Data Availability

The data that support the findings of this study are available from the corresponding author upon reasonable request.
