# Supplementary material for: Generation of Probabilistic Bits by Exploiting Orthogonal Spin Currents in Magnetic Trilayers
Source: Adv Sci (Weinh). 2026 Jul 23;13(58):e24371. doi: 10.1002/advs.202524371 (PMC13393264; doi:10.1002/advs.202524371)
Supplement: Supplementary file 1 [file ADVS-13-e24371-s001.pdf]

Supporting Information

Generation of probabilistic bits by exploiting orthogonal spin currents in magnetic trilayers

Donghyeon Han, Chaehyeon Shin, Seok-Jong Kim, Yunho Jang, Daekyu Koh, Taehwan Kim, Jaeheon Jung, Geon-Woo Baek, Minseok Kang, Eunseok Kim, Geun-Hee Lee, Jeongchun Ryu, Makoto Kohda, Junsaku Nitta, Kab-Jin Kim, Jongsun Park, Kyung-Jin Lee and Byong-Guk Park**

S1. Structural and magnetic characterization of an epitaxial Fe/Ti/CoFeB trilayer
S2. Spin-orbit torques in Ta/CoFeB/MgO
S3. Current-direction dependence of SOT switching.
S4. Stochastic switching in a polycrystalline trilayer
S5. Raw data of SOT switching measurements
S6. P-bit generation in nanosecond timescale
S7. Statistical test of random bit streams
S8. Initial state dependent probability
S9. Repetitive SOT switching experiment
S10. Domain wall depinning by *z*-spin polarized spin current
S11. Benchmarking against conventional CMOS RNGs
S12. Estimation of the device properties
S13. Reproducibility and device-to-device variation
S14. Current-dependent AHE loop shift in an Fe/Ti/CoFeB trilayer
S15. Derivation of the switching current density in tilayer
S16. LLG simulations of magnetization switching in a magnetic trilayer
S17. Forward AND gate operations
S18. P-bit streams with 16 different probabilities
S19. MAC operations of p-bit streams
S20. Comparison of energy and area consumption of stochastic neural networks
S21. Integer factorization and Max-Cut problems based on trilayer p-bits

1. Structural and magnetic characterization of an epitaxial Fe/Ti/CoFeB trilayer

Figure S1a shows the θ–2θ XRD pattern of the MgO sub./Fe (2 nm)/Ti (3 nm)/CoFeB (1 nm)/MgO (2 nm) heterostructure, where a clear Fe(200) diffraction peak is observed, indicating that the Fe(100) texture is well developed on MgO(001). In addition, Figures S1b and S1c show the in-plane magnetic hysteresis loops measured along the easy-axis directions, Fe[100] and Fe[010], and the hard-axis directions, Fe[110] and Fe[$1\bar{1}0$], respectively. The easy-axis loops exhibit square hysteresis with near-unity remanence, whereas the hard-axis loops show reduced remanence and gradual magnetization reversal. This clear difference is consistent with the fourfold cubic magnetic anisotropy expected for epitaxial Fe(001) films. Figure S1d shows an out-of-plane hysteresis curve, demonstrating perpendicular magnetic anisotropy of the top CoFeB layer.

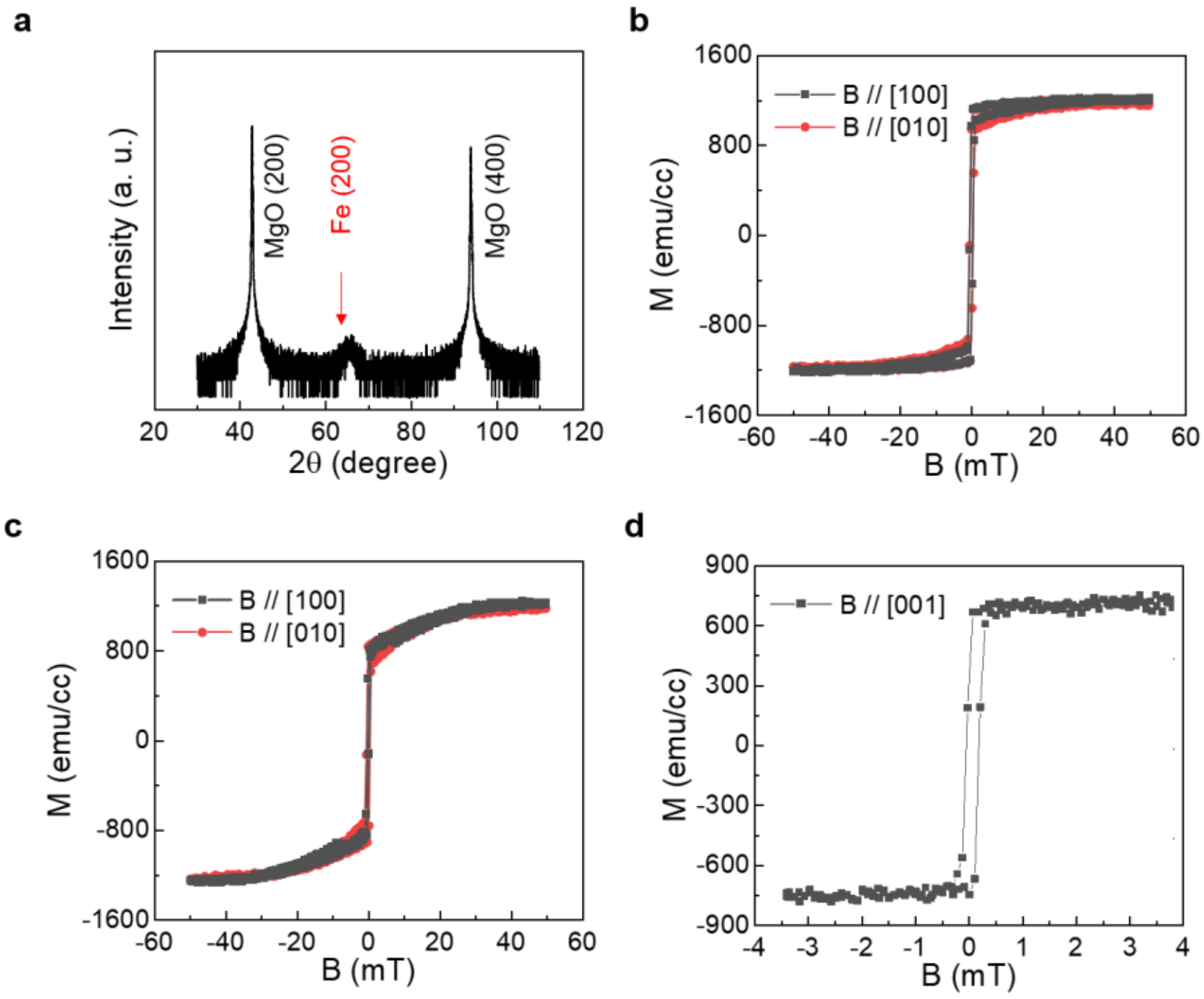

Figure S1. Structural and magnetic characteristics of an Fe/Ti/CoFeB trilayer. **a)** θ–2θ X-ray diffraction pattern of the trilayer. **b)** In-plane magnetic hysteresis loops measured along the easy-axis directions, Fe[100] and Fe[010]. **c)** In-plane magnetic hysteresis loops measured along the hard-axis directions, Fe[110] and Fe[$1\bar{1}0$]. **d)** Out-of-plane magnetic hysteresis loop of the CoFeB layer.

2. Spin-orbit torques in Ta/CoFeB/MgO

We investigated spin-orbit torque (SOT) in a control sample of a Ta(5 nm)/CoFeB(1 nm)/MgO(3 nm) structure. Figure S2 shows SOT switching with different in-plane magnetic fields (B_x). This demonstrates that deterministic SOT switching is observed for a B_x larger than 8 mT. Figure S3 shows AHE loop shift measurement results with d.c. currents $I_{d.c.} = \pm 3.5$ mA under a different B_x. The loop shift ΔB_S is absent at B_x = 0 mT, and it linearly increases with B_x when B_x is small and saturates at B_x = 90 mT.

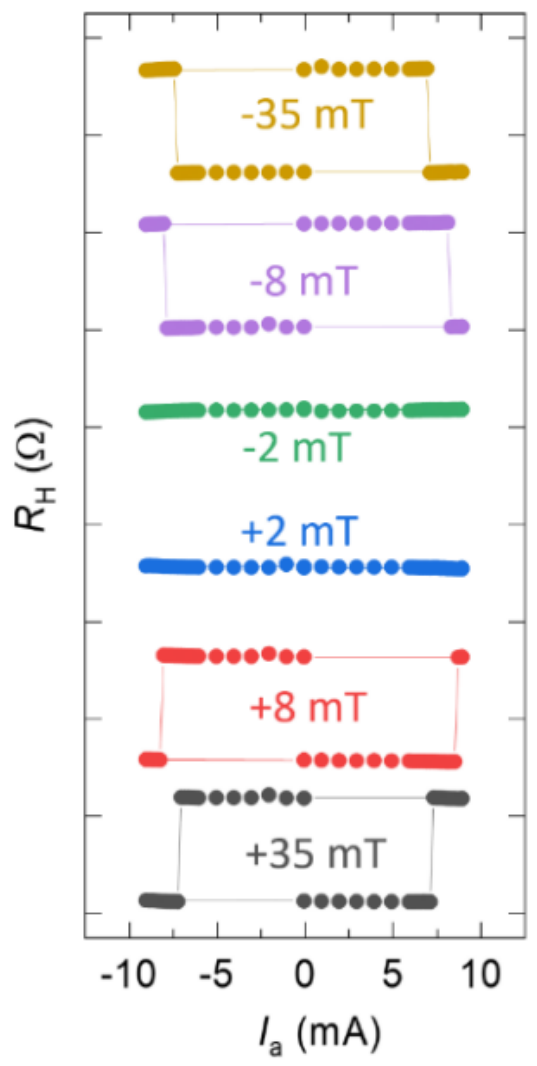

Figure S2. SOT switching in a Ta(5 nm)/CoFeB(1 nm)/MgO(3 nm) structure with different B_x.

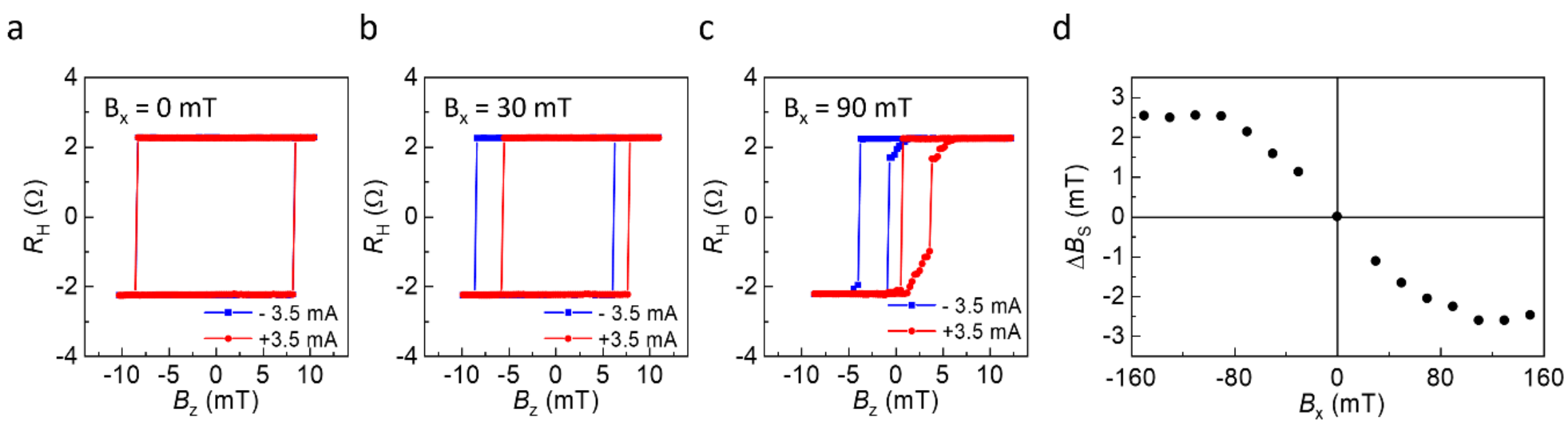

Figure S3. AHE loop shift in a Ta/CoFeB/MgO bilayer. **a-c)** AHE measurement results with d.c. current ± 3.5 mA under different B_x of 0 (a), 30 mT (b), and 90 mT (c). **d)** AHE loop shift ΔB_S versus B_x.

3. Current-direction dependence of SOT switching.

To investigate the role of the Fe magnetization orientation in the generation of orthogonal spin-current components, we performed SOT switching measurements with the current applied along different crystallographic directions of the epitaxial Fe layer. Figure S4 compares the SOT switching characteristics measured with the current applied along the Fe[100] easy-axis direction and the Fe[110] hard-axis direction.

When the current is applied along the Fe[100] easy axis (Figure S4a), clear SOT switching loops are observed under both a large in-plane magnetic field (20 mT) and a small magnetic field (~1 mT). The switching polarity observed at B_x ~ 1 mT is opposite to that observed at B_x ~ 20 mT, indicating the competition between the σ_y- and σ_z-polarized spin-current components. Under the large magnetic field, the switching behavior is primarily governed by the σ_y component assisted by the external field, whereas the low-field switching originates predominantly from the σ_z component generated through spin-orbit precession at the Fe/Ti interface.

In contrast, when the current is applied along the Fe[110] hard axis (Figure S4b), clear SOT switching is still observed under B_x ~ 20 mT. However, no distinct switching loop is observed under B_x ~ 1 mT. In this geometry, the Fe magnetization is rotated away from the current direction and tends to align toward the neighboring easy-axis directions. Consequently, the magnetization component parallel to the current direction (M_x) is substantially reduced, suppressing the generation of the σ_z-polarized spin current. As a result, the switching behavior observed at B_x ~ 1 mT. in the easy-axis is absent in the hard-axis geometry. We note that the signal fluctuations observed under B_x ~ 1 mT in the hard-axis geometry arise from the planar Hall effect associated with the reorientation of the Fe magnetization between the two neighboring easy-axis directions (±45° from the Fe[110] hard axis), rather than from current-induced switching of the CoFeB layer.

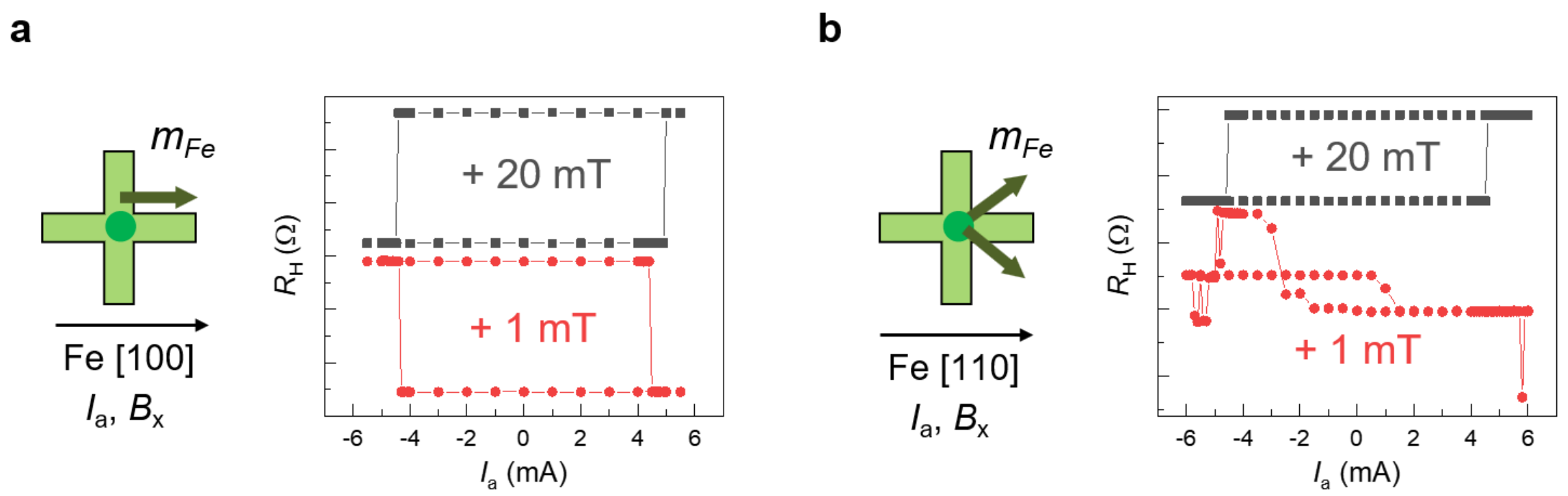

Figure S4| Crystallographic-direction dependence of SOT switching. a, SOT switching measured with current applied along the Fe[100] easy axis under in-plane magnetic fields of 1 and 20 mT. **b,** SOT switching measured with current applied along the Fe[110] hard axis under the same field conditions.

4. Stochastic switching in a polycrystalline trilayer

We investigated stochastic switching behavior in a polycrystalline trilayer consisting of an IrMn(15nm)/CoFeB(4nm)/Ta(3nm)/CoFeB(1nm)/MgO(2nm) structure grown on a thermally oxidized Si substrate (Figure S5a). In this structure, the bottom in-plane CoFeB is exchanged-coupled with the antiferromagnetic IrMn layer, while the top CoFeB layer exhibits perpendicular magnetic anisotropy. The samples were patterned into Hall bar devices with a width of 500 nm, featuring a 300 nm-sized CoFeB ferromagnetic island (Figure S5b). Figure S5c shows anomalous Hall resistance (R_H) as a function of the applied current (I_a) with different B_x values. At B_x = 10 mT, we observed deterministic switching with counter-clockwise polarity, while field-free deterministic switching occurs without applying B_x, but with the opposite polarity. For an intermediate B_x of 3 mT, the switching becomes stochastic. To confirm the stochastic switching in this sample, we also performed 100 switching trials using I_a of 6.6 mA while varying B_x (Figure S5d). At B_x = 0 mT, the SOT switching predominantly results in the magnetization being in the 'DOWN' state. With increasing B_x, the switching became stochastic, and the number of 'UP' states gradually increases. This is consistent with switching behavior observed in the epi-Fe trilayers, as shown in Figure 2b of the main text, confirming that stochastic switching can be observed in a polycrystalline sample. We note that the fabrication of polycrystalline p-bit devices was achieved below 700 K, which is the back-end compatible temperature of CMOS process.

We additionally investigated the switching behavior in a microscale device. Figure S6 shows repetitive SOT switching measurements obtained from a 1 μm × 1 μm IrMn/CoFeB/Ta/CoFeB/MgO device. Multiple intermediate Hall-resistance states are observed during the switching process, indicating multidomain formation. These results suggest that reducing the device dimensions suppresses multidomain formation and enables robust binary stochastic switching.

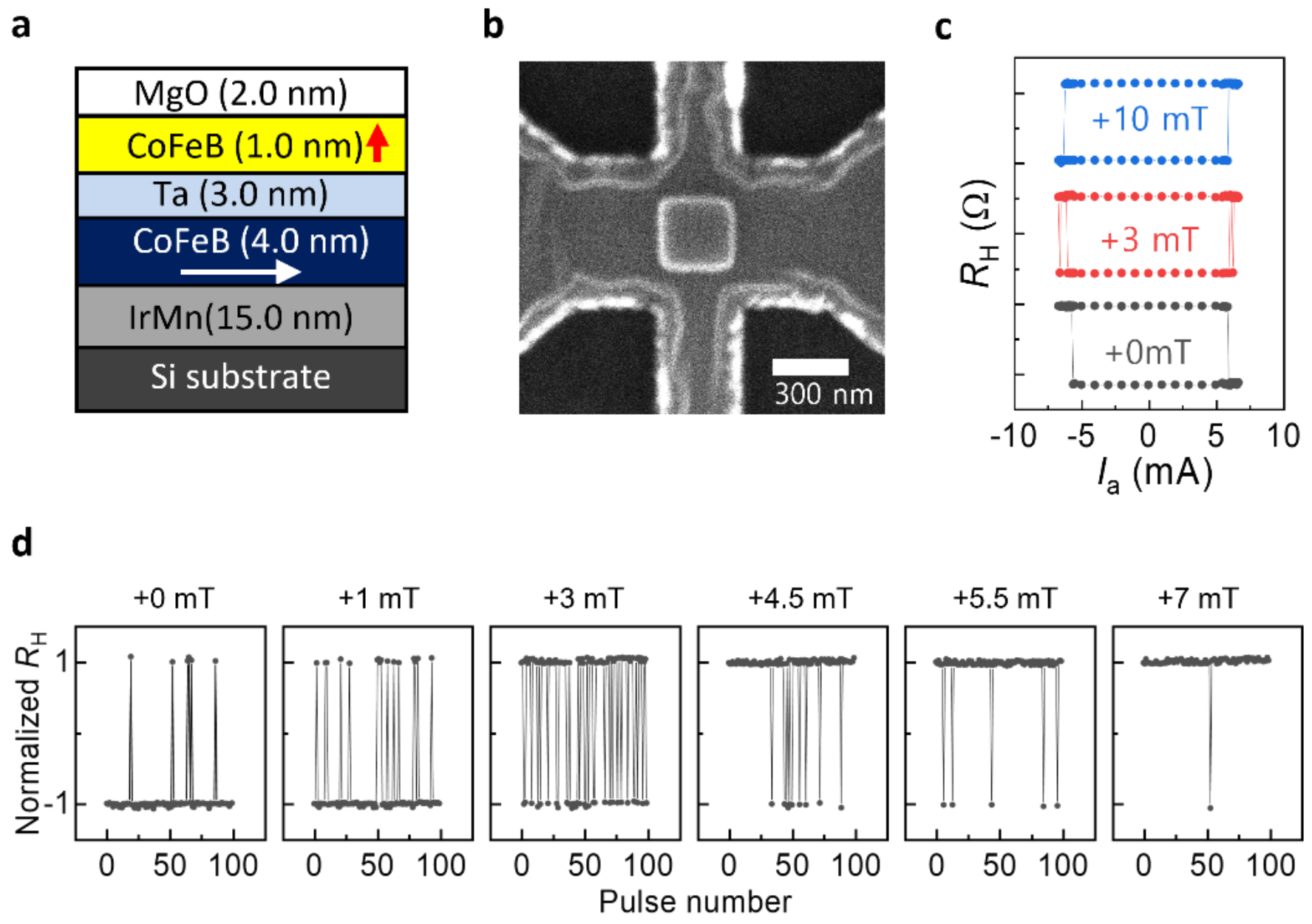

Figure S5. Stochastic SOT switching in poly-crystalline magnetic trilayer. a) Schematic illustration of the trilayer structure. **b)** SEM image of a Hall bar device. **c)** Current-induced SOT switching loops with or without in-plane magnetic field B_x. **d)** Repetitive SOT switching for 100 trials for different B_x with a constant I_a of 6.6 mA.

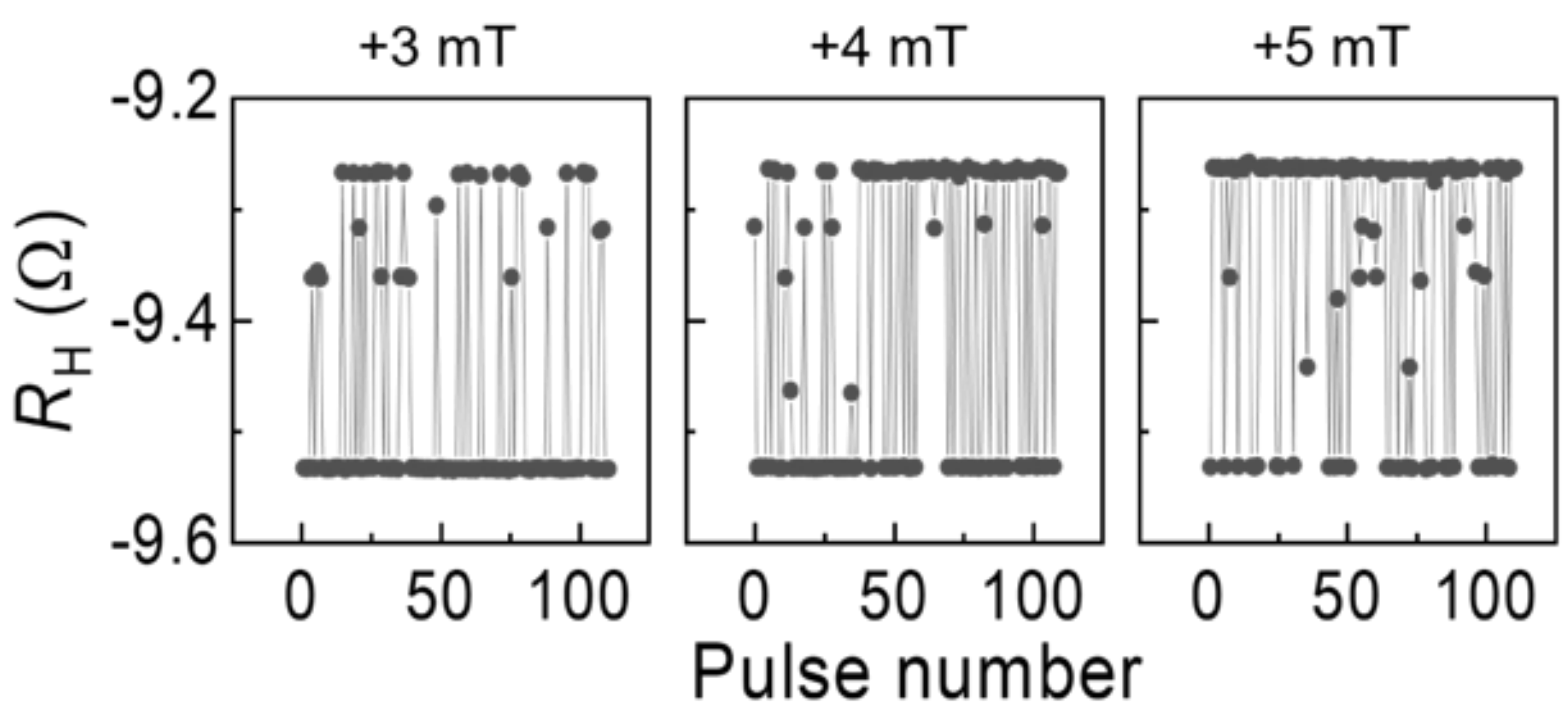

Figure S6. Stochastic SOT switching in a microscale polycrystalline trilayer. Repetitive SOT switching measurements obtained from a 1 μm × 1 μm IrMn/CoFeB/Ta/CoFeB/MgO device. A total of 110 consecutive switching events were measured under repeated current pulses.

5. Raw data of SOT switching measurements

Figure S7 shows raw data of Hall measurements corresponding to Figure 2a in the main text. Figure S7a shows the R_H as a function of out-of-plane magnetic field (B_z) in the epi-Fe/Ti/CoFeB trilayer. The blue dashed line indicates the remanent R_H values at the zero field, corresponding to the fully saturated 'UP' or 'DOWN' state of the perpendicular CoFeB layer. Figures S7b-f display the repetitive SOT switching results obtained from 1000 switching trials, where I_a is varied from 5.9 mA to 7.4 mA, while maintaining a constant B_x of 15 mT. The measured R_H values are confined to only two distinct resistance levels that indicated by the blue dashed lines, demonstrating that SOT fully switches the magnetization of the perpendicular CoFeB layer.

Figure S8 shows raw data of Hall measurements corresponding to Figure 2b in the main text, where the switching probability is controlled by varying the in-plane magnetic field. Figure S8a presents R_H as a function of out-of-plane magnetic field (B_z). Figures S8b-f display repetitive SOT switching results obtained from 1000 switching trials measured under repeated current pulses (I_a = 6.6 mA) at B_x = 1, 10, 14, 16, and 20 mT, respectively. Throughout the entire field range investigated, the measured (R_H) values remain confined to the two saturated resistance levels indicated by the blue dashed lines in Figure S8a, without observable intermediate states. These results demonstrate the current-induced Hall resistance change is essentially identical to the field-induced Hall resistance change, corresponding to a switching ratio of nearly 100%. The absence of intermediate Hall resistance states throughout the p-bit operating field range provides experimental evidence that domain-wall pinning and multi-domain formation are strongly suppressed during the switching process in the epi-Fe/Ti/CoFeB structures.

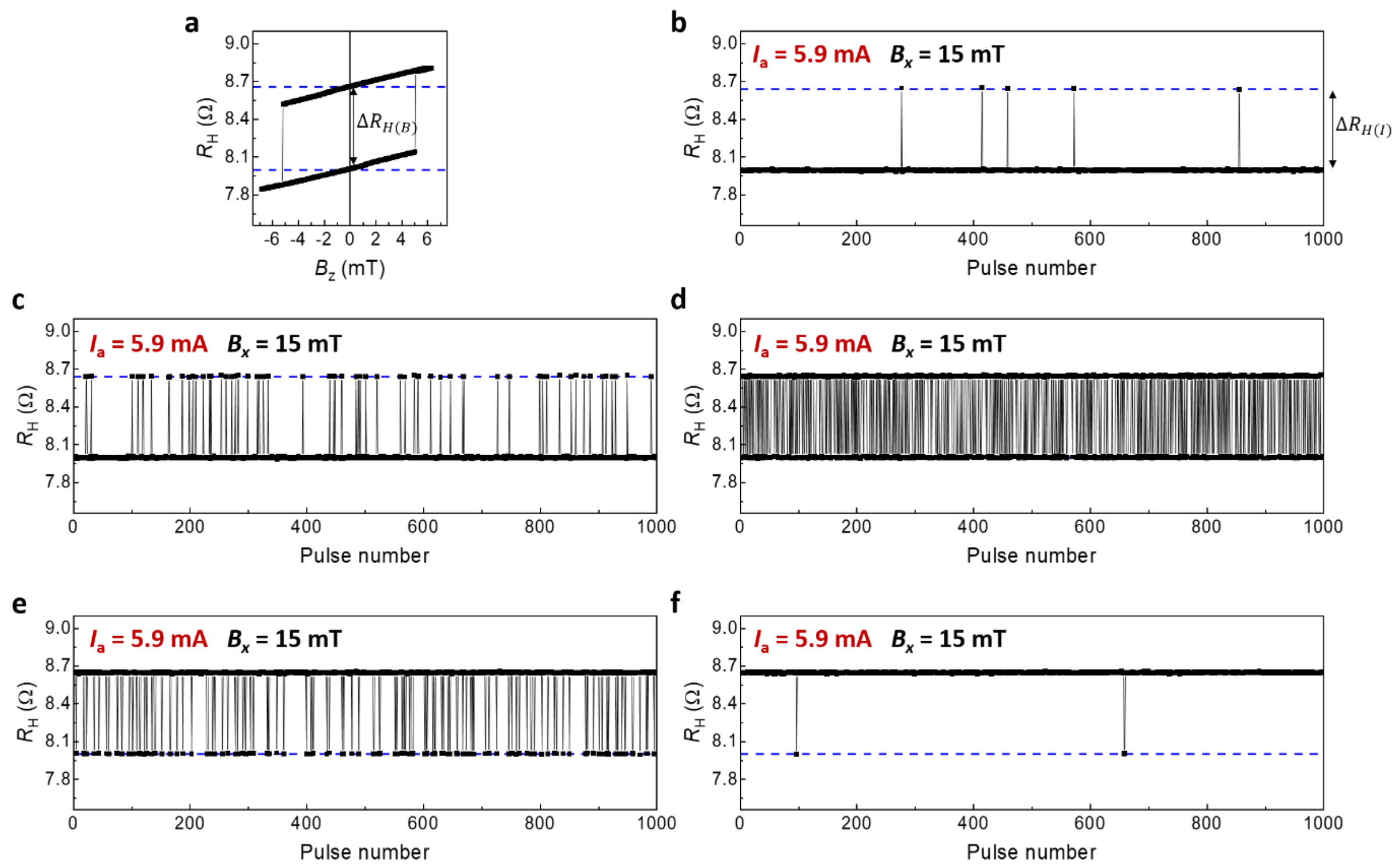

Figure S7. Raw data of AHE and SOT switching results. a) AHE loop. **b-f)** Repetitive SOT switching for 1000 trials for different I_a of 5.9 mA (b), 6.2 mA (c), 6.5 mA (d), 6.7 mA (e), and 7.2 mA (f) with a constant B_x of 15 mT.

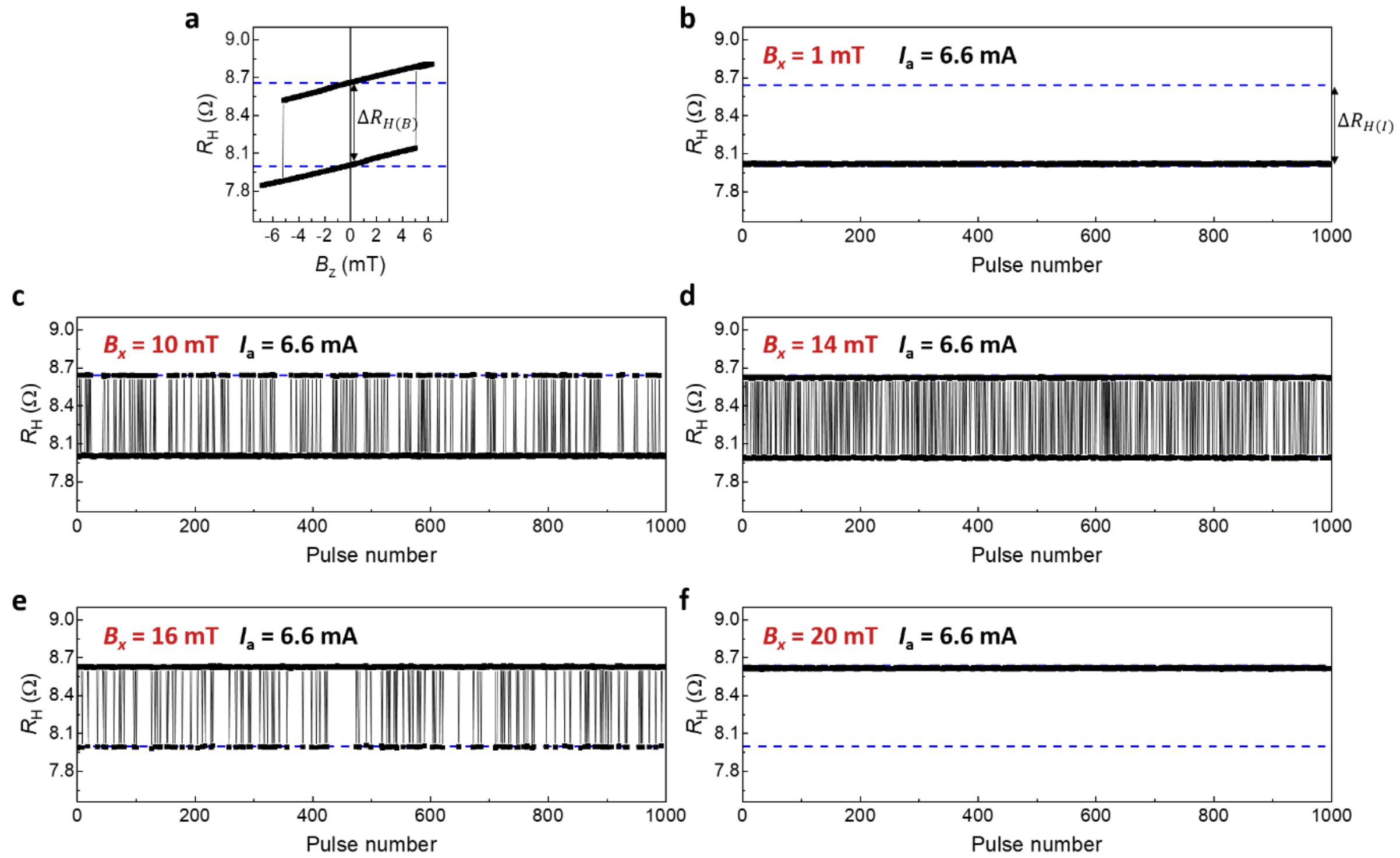

Figure S8. Raw data of AHE and SOT switching results. a) AHE loop. **b-f)** Repetitive SOT switching for 1000 trials for different B_x of 1 mT (b), 10 mT (c), 14 mT (d), 16 mT (e), and 20 mT (f) with a constant I_a of 6.6 mA.

6. P-bit generation in nanosecond timescale

We examined the timescale of probabilistic switching in our device using additional experiments and micromagnetic simulations. First, we conducted additional experiments using a nanosecond current pulse using a polycrystalline trilayer of IrMn(15 nm)/CoFeB(4 nm)/Ta(3 nm)/CoFeB(1 nm)/MgO(2 nm), featuring a 300 nm ferromagnetic CoFeB island (Figure S9a,b). Figure S9c illustrates the measurement setup, where a pulse generator applies a pulsed current (I_a) via a bias tee, while a DC current is used to measure the Hall resistance. Figure S9d shows the 10 ns pulse shape used in the switching measurement. To test stochastic switching, we performed 110 switching trials using a 10 ns I_a of 6.6 mA while varying in-plane magnetic field, B_x (Figure S9e). At B_x = 0 mT, SOT switching predominantly results in the magnetization being in the 'DOWN' state. As B_x increases, switching becomes stochastic, and the number of 'UP' states gradually increases, demonstrating the switching probability is controlled by the combination of I_a and B_x. Figure S9f shows the P_{UP} as function of B_x, exhibiting a sigmoid curve, consistent with the results obtained using a longer current pulse (Figure 2 in the original manuscript). Figure S9g presents 2200 repeated switching cycles under B_x = 6 mT, confirming reliable p-bit generation on the nanosecond timescale.

Second, we performed micromagnetic simulations of SOT-induced switching dynamics. Figure S10 shows the time evolution of the z-component of the magnetization (m_z), which was initially aligned along the +z direction. Upon applying a 0.1 ns current pulse (Figure S10a), the magnetization rapidly rotates toward the saddle point, where m_z is zero. The inset in Figure S10b highlights the early-stage dynamics, showing that the magnetization reaches the saddle point at approximately 50 ps after the current pulse. Although the exact dynamics depend on the applied current density, the transition typically occurs within 100 ps. This confirms that the 0.14 ns current pulse used in our bit generation energy calculation is an adequate time to drive the magnetization to the saddle point. Importantly, this process occurs regardless of the initial magnetization directions, as shown in Figure S10c, where the magnetization reaches the saddle

point within approximately 50 ps irrespective of its initial state. However, after the pulse current is turned off, the magnetization relaxes to either the UP or DOWN state within 1~10 ns, depending on the damping constant. This relaxation time determines the bit-generation speed, which is longer than 0.14 ns, but comparable to that of s-MTJ devices.

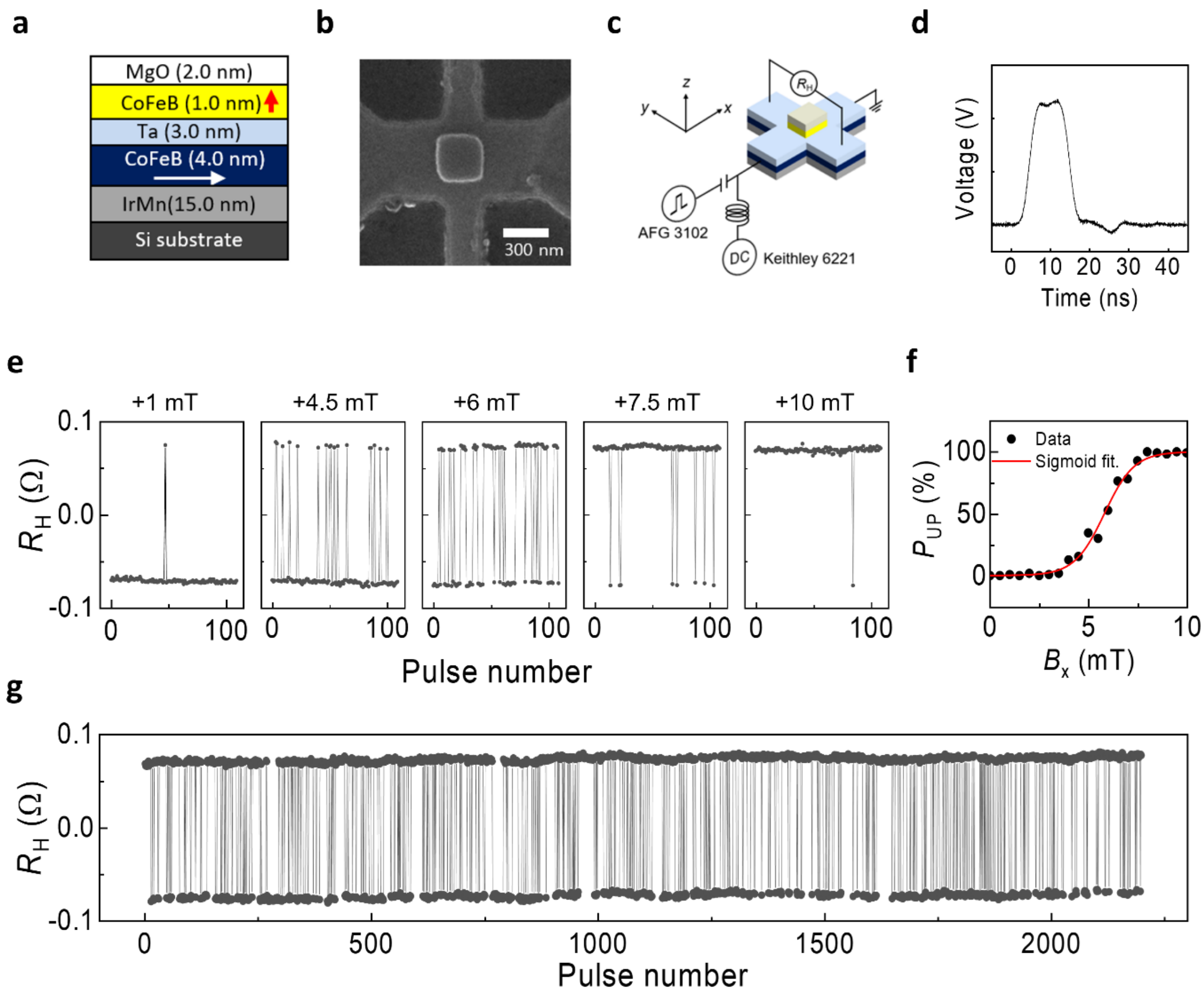

Figure S9. Nanosecond p-bit generation in poly-crystalline magnetic trilayer. a) Schematic illustration of the trilayer structure. **b)** SEM image of a Hall bar device. **c)** Schematic illustration of the measurement set up. **d)** Shape of pulse measured in oscilloscope. **e)** Repetitive SOT switching for 110 trials for different B_x with a constant I_a of 8.3 mA. **f)** P_{UP} as function of B_x. The red curves indicate the sigmoid fitting. **g)** Repetitive SOT switching for 2200 trials for B_x = 6 mT with a constant I_a of 8.3 mA

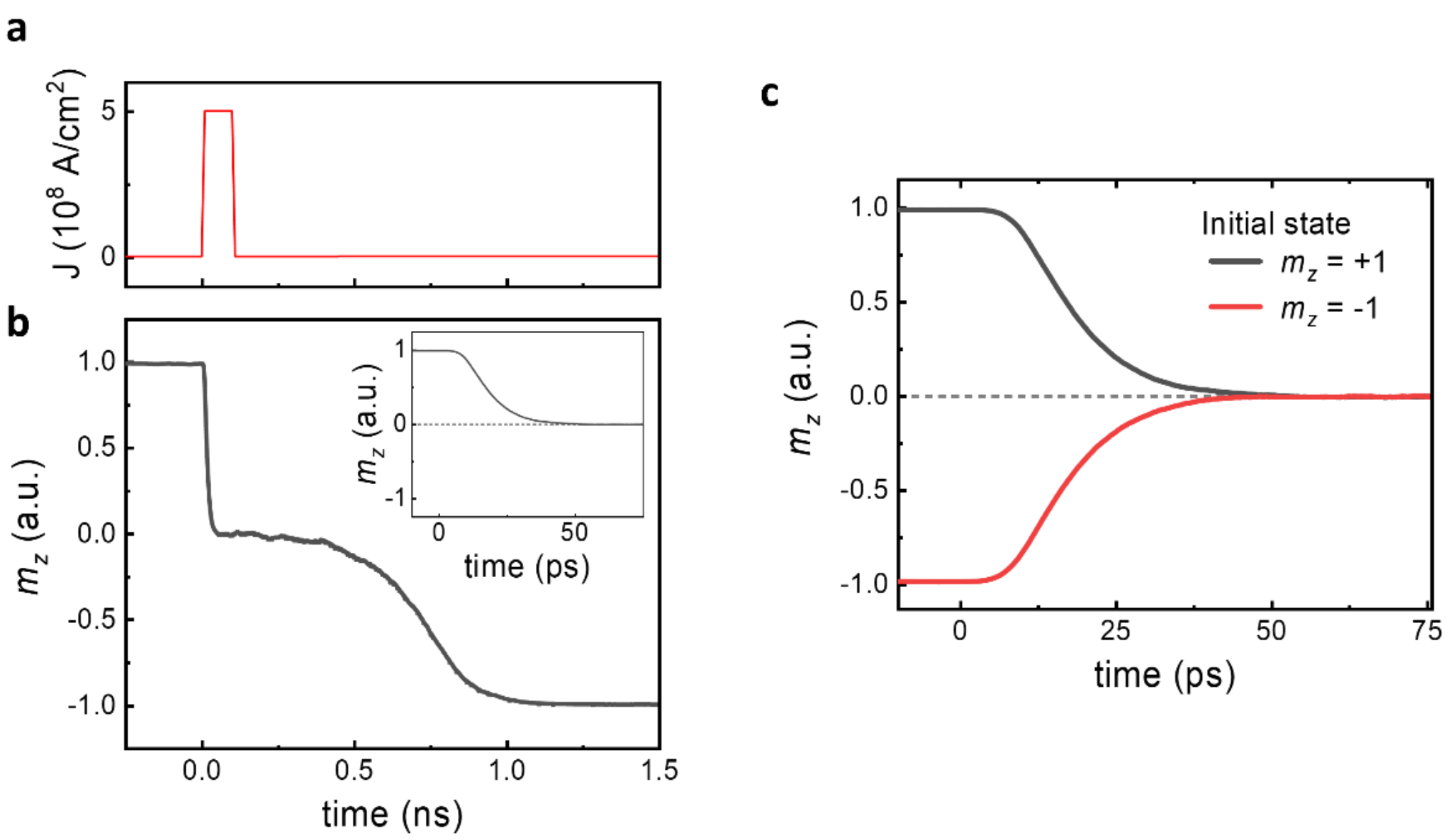

Figure S10. SOT switching calculation results. a) Applied current density. **b)** Trajectory of m_z component SOT switching. Inset zooms in on the initial magnetization movement toward the saddle point after pulse application. **c)** Time evolution of the *z*-component when current is applied at 0 ns for initial state $\pm m_z$. Parameters used for the simulations are as follows: $B_k = 0.2$ T, $M_S = 500$ kA/m, $T = 300$ K, $V = 500 \times 500 \times 1$ nm^3, $\alpha = 0.2$, $\theta_{SH,D} = 0.2$, $t_F = 1$ nm, current pulse width = 0.1 ns.

7. Statistical test of random bit streams

We investigated statistical properties of our random bit. We first generated a random bit stream with a length of 100,000 (Figure S11a). To obtain random bits (P_{UP} = 50 %), we set the applying a current of 6.5 mA and a magnetic field of 12.3 mT. The results of P_{UP} = 50.134 %. Using this random bit stream, we calculated autocorrelation function based on $r_k = \frac{\sum_{i=1}^{n-k}(x_i-\bar{x})(x_{i+k}-\bar{x})}{\sum_{i=1}^{n}(x_i-\bar{x})^2}$ (Figure S11b). The autocorrelation function remains within the range of $\pm$ 0.02, indicating the independence of bits. Figure S11c shows the standard deviation of autocorrelation, which follows a $1/\sqrt{N}$ trend, consistent with the binomial random distribution.

For the NIST statistical tests (SP 800-22)[S1], we extended the measurement to a 1.1 million-bit stream. We found that the probability in the raw streams shown in Figure S12a,b slightly deviated from the ideal 50% value, which might be due to long acquisition time of ~50 hours. To mitigate this effect[S2], we applied an XOR operation between two independently measured 1.1 million-bit streams with similar bias (P_{UP}= 49.14 and P_{UP} = 49.09%, Figure S12a and b, respectively). The XOR-ed stream exhibited P_1= 49.97% (Figure S12c) and successfully passed all 15 NIST tests (Table S1).

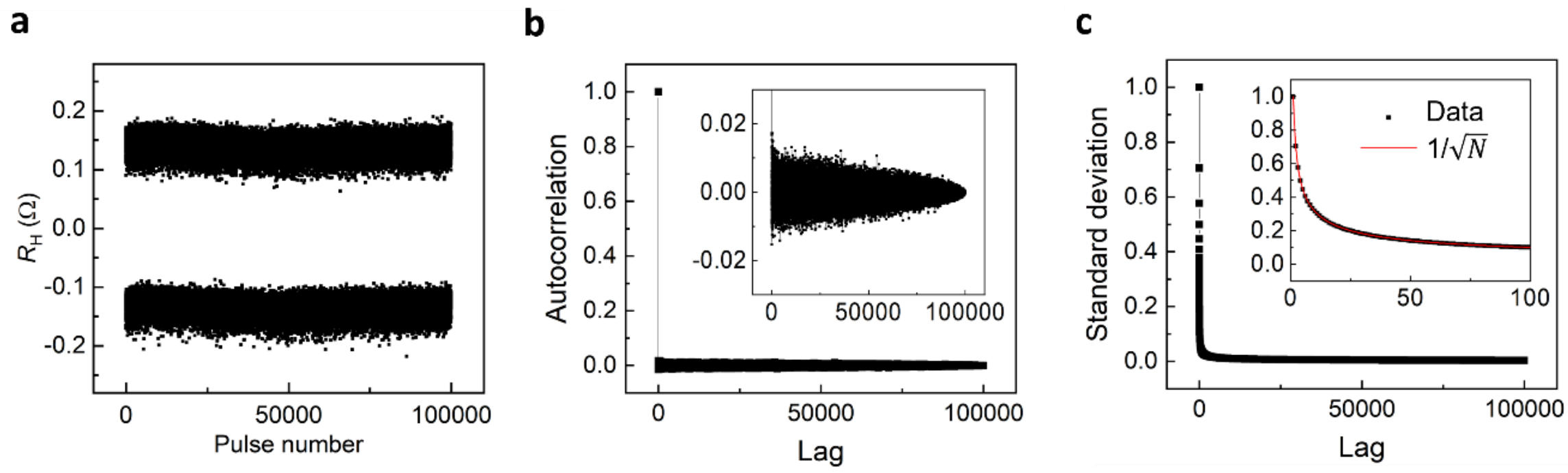

Figure S11. Statistical properties of random bit stream. a) Generated random bit stream. **b)** Autocorrelation function of generated bit stream. **c)** Standard deviation of autocorrelation. (The insets are magnified image)

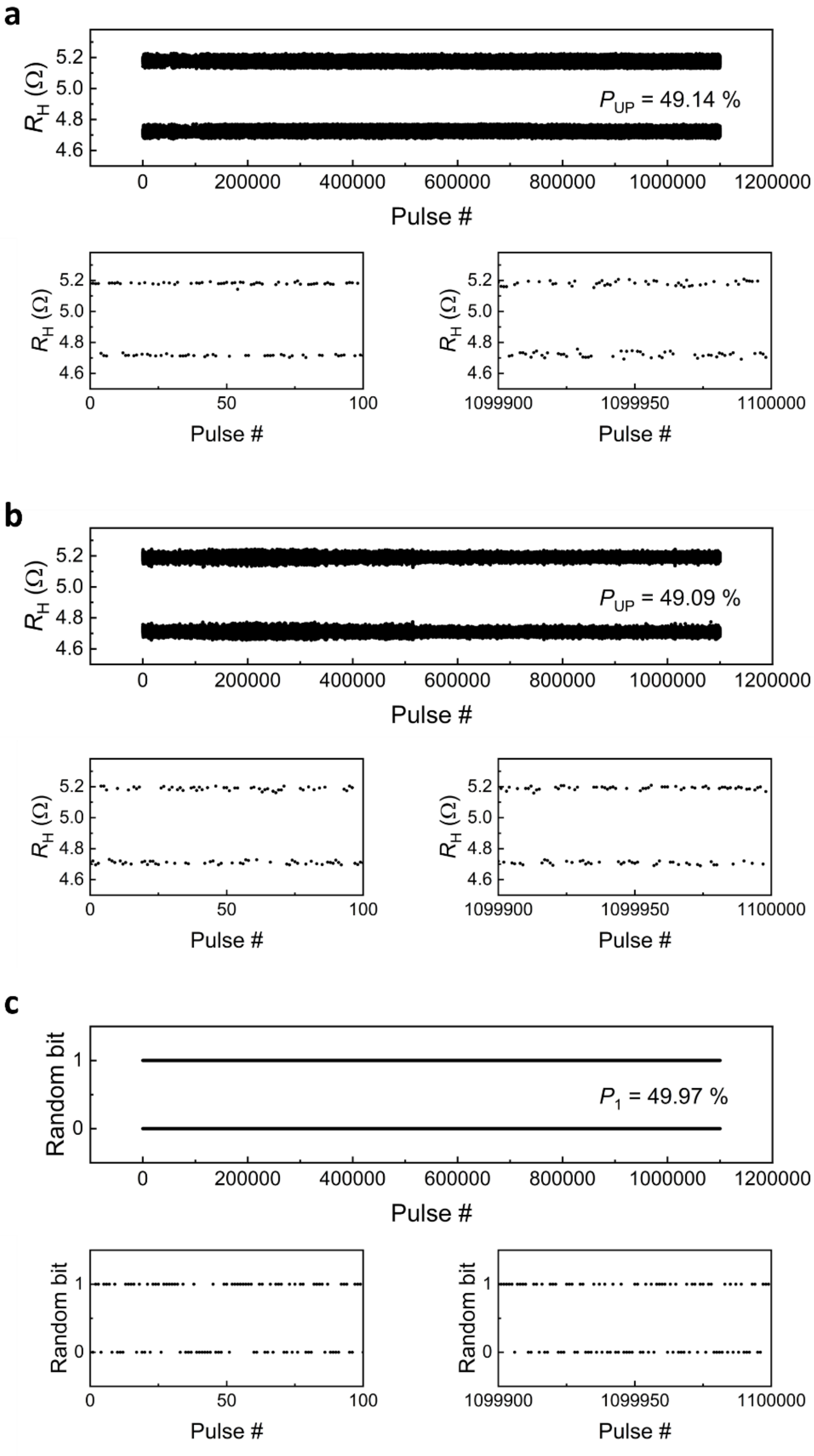

Figure S12. Random bit streams of 1.1-million-bits a) Measured R_H with P_{UP} = 49.14 %. **b)** Measured R_H P_{UP} = 49.09 %. **c)** Random bit stream by XOR operation of a and b.

Table S1. NIST statistical test results

Number	Test Name	P-value	Result
1	Frequency	0.538	success
2	Block frequency	0.478	success
3	Runs	0.177	success
4	Longest run	0.105	success
5	Binary matrix rank	0.420	success
6	Discrete Fourier transform	0.903	success
7	Nonoverlapping template matching	0.520	success
8	Overlapping template matching	0.999	success
9	Linear complexity	0.615	success
10	Serial	0.438	success
11	Approximate entropy	0.848	success
12	Cumulative sum	0.319	success
13	Universal	0.146	success
14	Random excursions	0.550	success
15	Random excursions Variant	0.557	success

8. Initial state dependent probability

To explicitly demonstrate initial-state independence, we further analyzed the raw bitstream data shown in Fig. S26 by categorizing switching events according to the magnetization state immediately before each current pulse, while maintaining identical current and magnetic-field conditions. The results are summarized in Fig. S13, which shows that the switching probabilities for UP and DOWN initial states are statistically identical within experimental uncertainty. This provides direct experimental evidence that the probabilistic behavior is independent of the initial state, thereby confirming the initialization-free operation.

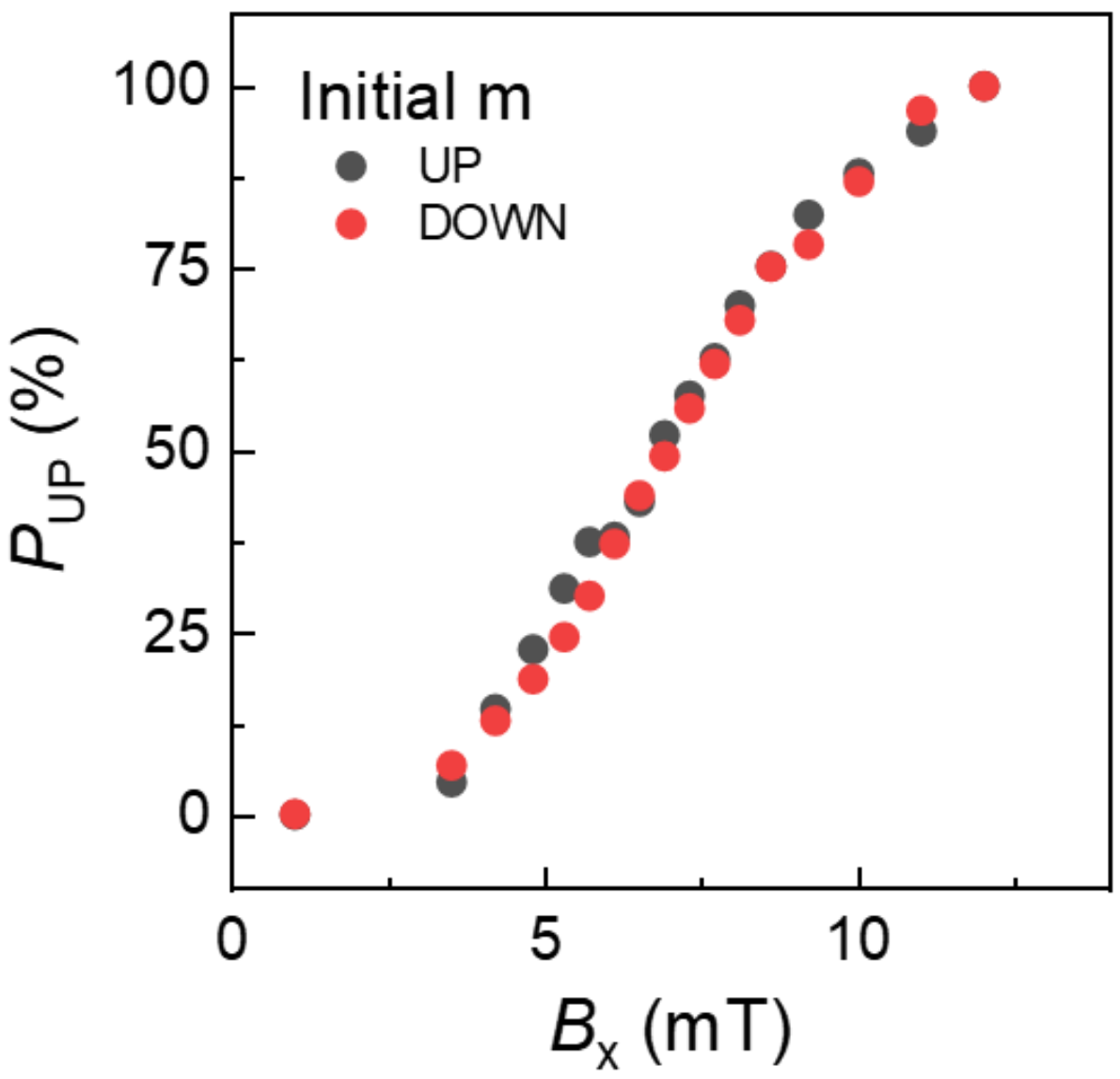

Figure S13. Initial state dependent P_{UP} as a function of B_x

9. Repetitive SOT switching experiment

We conducted repetitive switching experiments with applying a current of 7 mA and an in-plane magnetic field of 7.3 mT. Figure S14 shows the results of one million switching iterations. During iterations, the Hall resistance shows binary states without any intermediate state.

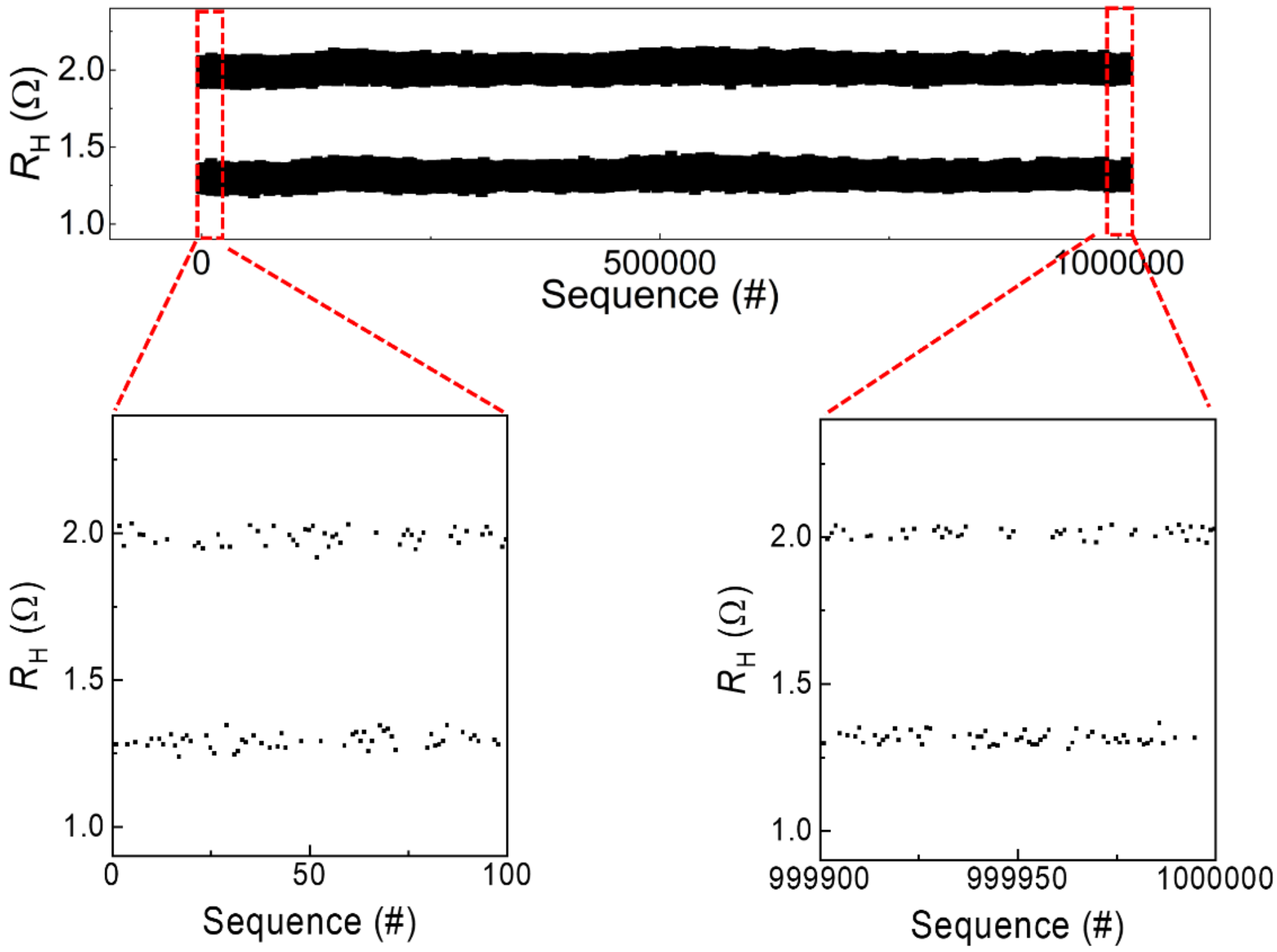

Figure S14. One million repetitive switching experiment results.

10. Domain wall depinning by *z*-spin polarized spin current

To investigate single-domain-like switching behavior in epi-Fe trilayer, we performed numerical simulation in a 1-dimensional ferromagnetic chain with an anisotropy defect (Figure S15a). During SOT switching, the defect can act as a domain wall pinning site. We defined the domain wall depinning current density J_D as a minimum current for domain wall depinning. Figure S15b shows the domain wall depinning current density as a function of η, where $\tan\eta = \sigma_z/\sigma_y$. As η decreases, (*z*-spin/*y*-spin ratio increases), the J_D decreases sharply. This implies that a high ratio of *z*-spin/*y*-spin assists the domain wall depinning during the SOT switching resulting in single-domain-like switching without forming multi-domain.

Figure S16 shows the numerical simulation results of SOT switching in a 400×400 nm^2 square sample with different η values. We initialized the magnetization to the "UP" direction (+ m_z), and then applied the SOT current for 3 ns and waited 10 ns for the domain structure to stabilize. Figures S16a-d show the domain patterns after SOT switching for different η values. For η = 180 (pure y-spin), the pattern exhibits a mixture of "UP" and "DOWN" domains. As the η value decreases (*z*-spin contribution increases), the "DOWN" domain portion increases and reaches a single "DOWN" domain at η = 159°. This suggests that a low sin η value, or a large z-spin contribution, favors single-domain-like switching, enabling reliable p-bit generation.

These simulation results are consistent with the following two experimental observations. First, the epi-Fe trilayer exhibits complete field-free SOT switching. Figure S17 compares the Hall resistance change obtained from field-sweep anomalous Hall measurements (Figure S17a) with that obtained from current-induced SOT switching measurements (Figure S17b). The measured $\Delta R_{H(I)}$ is essentially identical to $\Delta R_{H(B)}$, corresponding to a switching ratio [$\Delta R_{H(I)}/\Delta R_{H(B)}$] of approximately 99%. This result confirms that the current-induced switching occurs between the fully saturated 'UP' and 'DOWN' magnetization states. Second, SOT

switching occurs without intermediate Hall resistance states, as shown in Supporting Information 5 and 9. This observation suggests that domain-wall pinning and multi-domain formation are strongly suppressed during the switching process.

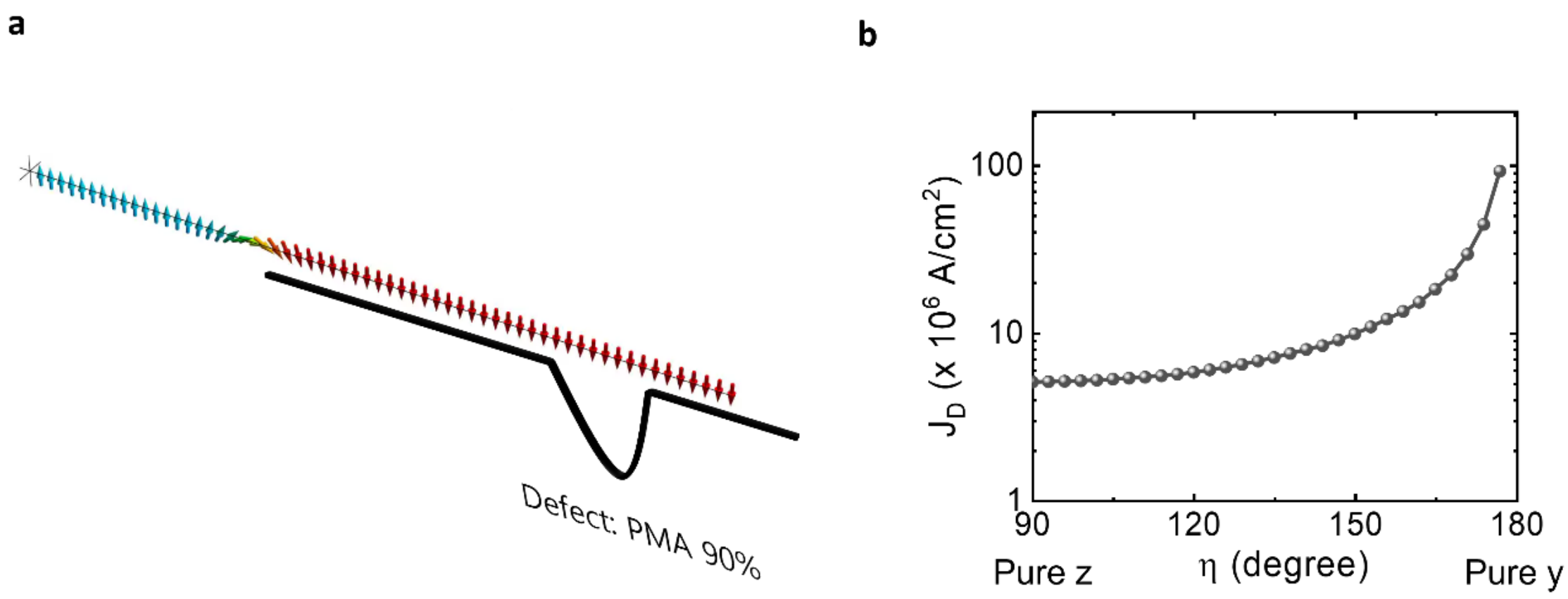

Figure S15. Numerical simulation results. a) 1-dimensional ferromagnet chain with an anisotropy defect. **b)** η-dependent domain wall depinning current J_D.

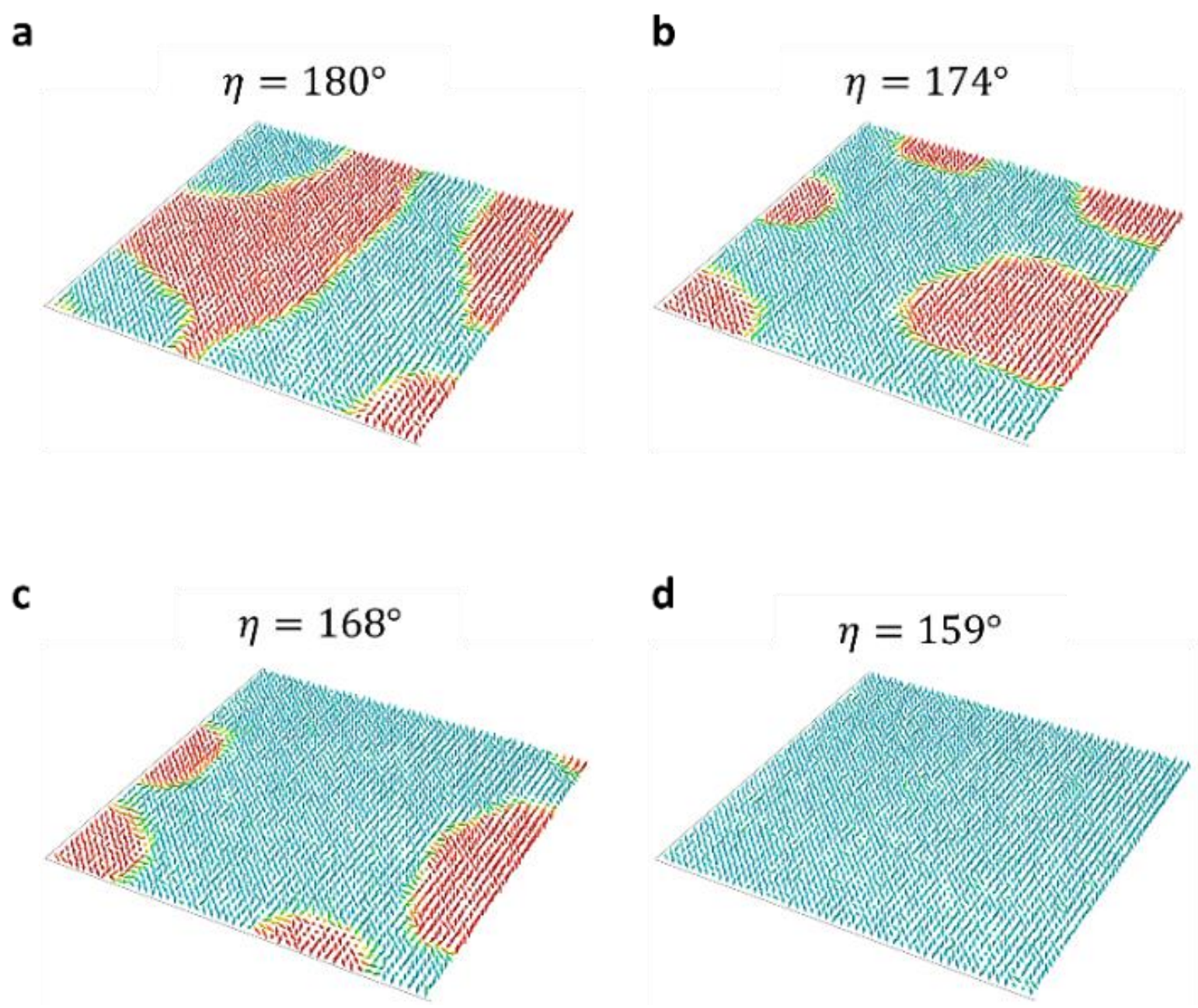

Figure S16. Numerical simulation results. a-d) Domain pattern after SOT switching. (a) $\eta = 180°$ (b) $\eta = 174°$ (c) $\eta = 168°$ (d) $\eta = 159°$.

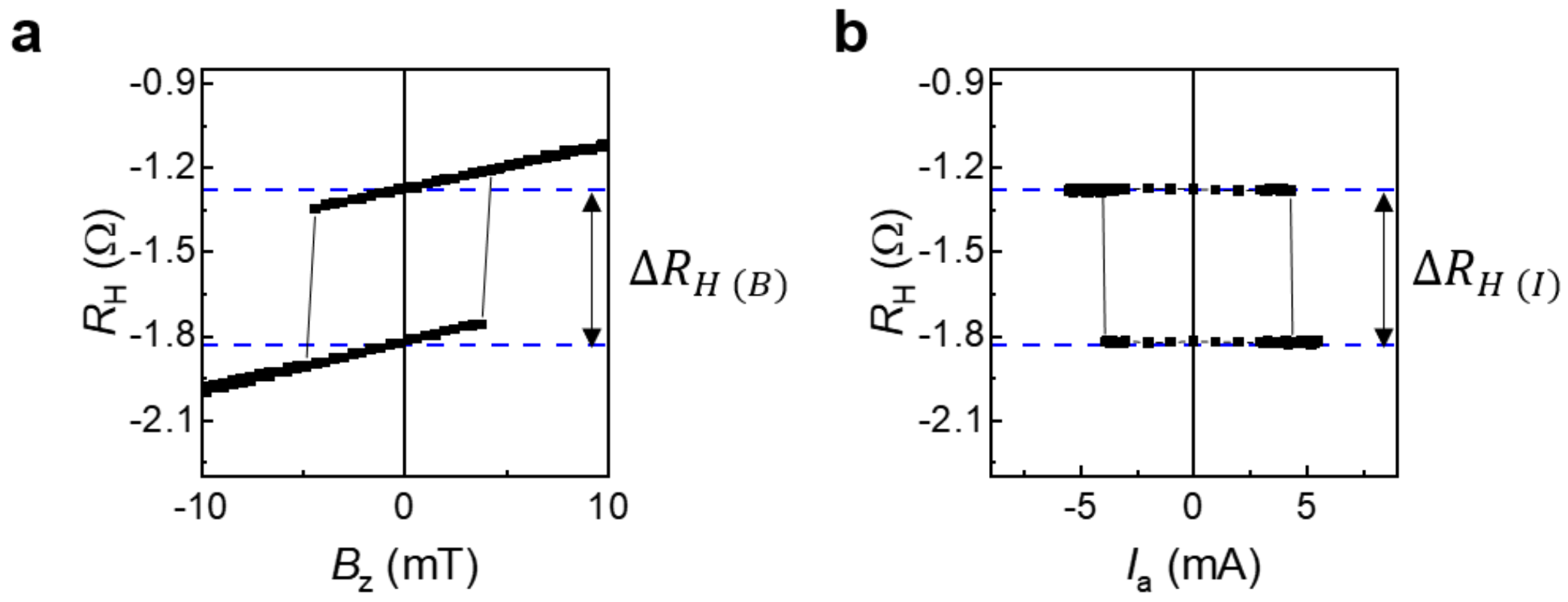

Figure S17. Evaluation of the SOT-induced switching ratio at B_x = 0 mT. a, Anomalous Hall resistance changes obtained from perpendicular field sweep measurements ($\Delta R_{H(B)}$). **b,** Anomalous Hall resistance change obtained from current-induced switching measurement at B_x = 0 mT ($\Delta R_{H(I)}$)

11. Benchmarking against conventional CMOS RNGs

The energy consumption and area for random-bit (RB) generation were benchmarked against representative CMOS-based random number generators (RNGs) reported in the literature: (i) a ring-oscillator-based true random number generator (TRNG)[S3] and (ii) a ring-oscillator-based RNG[S4]. Both implementations were realized in a 28-nm CMOS technology node and represent widely adopted RNG architectures.

From the reported data, the RB generation energy and core area were extracted as 23 pJ/bit and 375 μm² for[S3], and 1.1 pJ/bit and 3090 μm² for[S4], respectively. The former is a TRNG with experimentally verified randomness quality, whereas the latter represents a highly optimized CMOS-based RNG implementation. Both designs operate at comparable throughput on the order of tens of megabits per second (~23 Mbit/s and ~25 MHz operation, respectively).

For the trilayer p-bit, a magnetic tunnel junction (MTJ) structure was assumed, in which the perpendicular magnetization state of CoFeB in the trilayer serves as the free layer of the MTJ. The simulation parameters are summarized in Table S2, and the corresponding MTJ circuit, including 28 nm CMOS access transistors, is shown in Fig. S18. Based on these parameters, the energy consumption was estimated using SPICE simulations based on an SOT-MTJ MRAM circuit model adopted from[S5], which provides a comparative and experimentally validated SPICE framework for spin-transfer-torque and spin-Hall-effect switching mechanisms in perpendicular MTJs. Including both write and read operations, the total energy consumption for probabilistic bit generation is calculated to be 0.209 pJ/bit, with a corresponding cell area of 1.896 μm².

The results are summarized in Table S3. Using these parameters, the trilayer p-bit device exhibits more than 100 (5)× lower energy consumption and more than 200 (1500)× smaller area compared to the CMOS-based true (pseudo) RNGs.

Table S2. Simulation parameters for the trilayer MTJ

Parameter	Value
Thickness of free layer	1 nm
Writing path dimension	40 nm × 80 nm × 5 nm
Writing path resistivity	250 $\mu\Omega\cdot$cm
MTJ dimension	30 nm × 30 nm
RA	5 $\Omega\mu m^2$
TMR	150 % (R_{OFF}/R_{ON} ratio =2.5)
MTJ resistance	5.6 kΩ (Parallel), 13.9 kΩ (Anti-parallel)
Random bit generation current density	100 MA/cm^2
Random bit generation current pulse width	1 ns

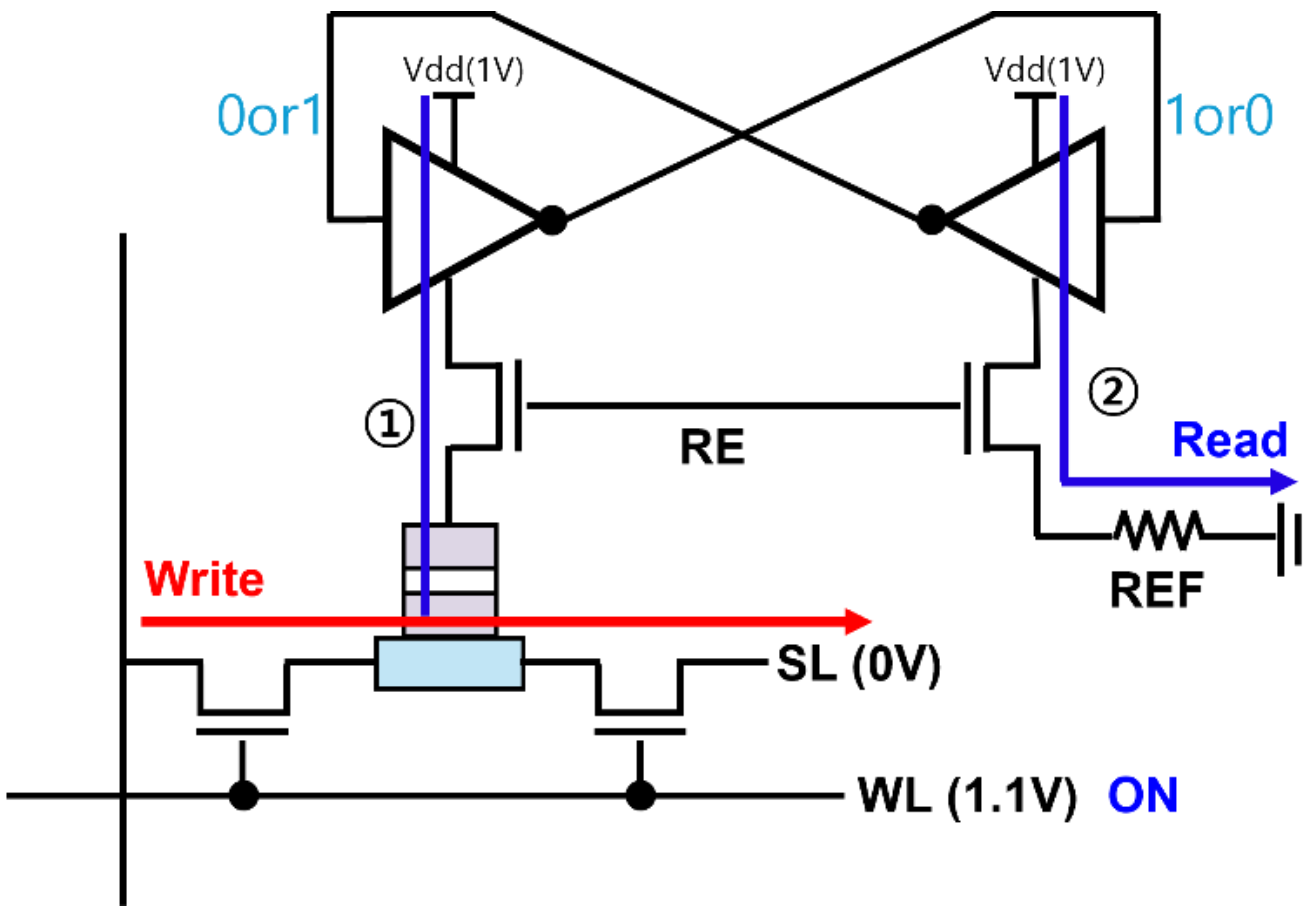

Figure S18. Schematic illustration of the SPICE simulation model.

Table S3. Core-level energy and area comparison between the trilayer p-bit and representative 28-nm CMOS RNGs

Architecture	Technology Node	Energy per Bit	Core Area	Throughput
CMOS-TRNG[S1]	28 nm	23 pJ	375 μm^2	23 Mbit/s
CMOS-PRNG[S2]	28 nm	1.1 pJ	3090 μm^2	25 Mbit/s
Trilayer p-bit	Magnetic trilayer	0.209 pJ	1.896 μm^2	Sub-ns

12. Estimation of the device properties

We estimated the device properties of our proposed trilayer p-bit compared to the conventional stochastic MTJs (s-MTJ) in terms of energy consumption, speed, and reliability. First, the bit generation energy of s-MTJs was estimated to be 2 fJ[S6], assuming an autocorrelation time of 100 ps. However, given these relaxation time is milliseconds for perpendicular-MTJs [S6] and 8 ns for in-plane nanomagnets[S7], the bit generation energy would be much larger than 2 fJ.

In trilayer p-bits, the bit generation energy is determined by SOT switching energy. To estimate the bit generation energy, we assumed a nanoscale implementation of the proposed p-bit device. This assumption enables a fair comparison with previously reported nanoscale s-MTJs. For this purpose, we refer to experimentally measured switching parameters from our previous work on nanoscale CoFeB/Ti/CoFeB trilayer devices[S8], where the field-free SOT switching current density was ~7×10^7 A/cm^2 and the writing pulse width was 0.14 ns. Based on these values, we modeled an MRAM circuit based on SOT-MTJ architecture, as illustrated in Figure S14. For the write operation (Figure S19a), a voltage of 1.5 V is applied to the word line (WL) to activate the access transistor, while a voltage of 0.261 V is applied to the bit line (BL) to drive the required current density for 0.14 ns. The energy consumed during this pulse was calculated using SPICE simulations, yielding a write energy of 5.5 fJ per bit.

For the read operation (Figure S19b), the WL is activated to enable access to the MRAM cell, and the read enable signal (RE) is asserted to turn on the switch that connects the MRAM cell to the cross-coupled inverter-based sensing circuit. The BL and source line (SL) are grounded to establish the appropriate biasing conditions. The MRAM cell stores either a logical ‘0’ or ‘1’, corresponding to the low or high resistance state of the magnetic tunnel junction (MTJ). The resistance difference results in a variation in the current through the MRAM cell (labeled as path ①), while a reference current flows through a fixed reference resistor (path ②). Depending on the relative magnitudes of these currents, the charging and discharging

behavior at the inverter nodes differs, ultimately identifying the stored logic state. The energy required for this read operation was calculated using SPICE simulations, yielding a read energy of 0.7 fJ per bit. Including both write and read operations, the total energy per bit is 6.2 fJ.

We note that the bit generation energy could be further reduced by decreasing the switching current density and/or the pulse width. Figure S20 presents the estimated bit generation energy as a function of the SOT switching current density at a pulse width of 0.14 ns. This suggests that the energy consumption of our trilayer p-bits device could potentially be lower than that of the s-MTJs, indicated by the red dashed line.

Second, we discuss the bit generation speed, another important factor of p-bit devices. For s-MTJs, the bit generation speed is limited by the relaxation time of thermal fluctuations, as the generation time (sampling time) must exceed the relaxation time to ensure reliable p-bit generation. On the other hand, trilayer p-bit device leverages competing SOTs rather than the thermal fluctuations of magnetization. Therefore, the bit generation speed is determined by the SOT switching, which has been demonstrated to be as fast as sub-nanosecond in several studies[S9,S10], including our previous work[S8]. Notably, recent work has demonstrated SOT switching with a switching time of 70 ps in Ta/Pt/Co/Cu/Ta multilayer with an assist field of 160 mT[S11]. Thus, the bit generation speed in our p-bit device, independent of relaxation time, can be faster than that of s-MTJ.

Lastly, our device generates p-bit through two competing torques, eliminating the need to reduce device dimensions into the superparamagnetic regime. The energy barrier of the free layer does not need to approach the low kBT, making it more robust to external factors. In contrast, s-MTJ can be sensitive to the external factor because the p-bit generation relies on thermal fluctuations. The relaxation time of thermal fluctuations is given by $\tau_{relax} \propto \exp(\frac{K_{\mathrm{eff}}V}{k_B T})$, where K_{eff}, V, k_B, and T are effective anisotropy constant, free layer volume,

Boltzmann constant, and temperature, respectively. Thus, the p-bit device based on s-MTJs is affected by the variation of temperature and device size.

Therefore, compared to s-MTJs, our p-bit device offers the potential for similar or even superior energy efficiency, faster bit generation speeds, and enhanced robustness.

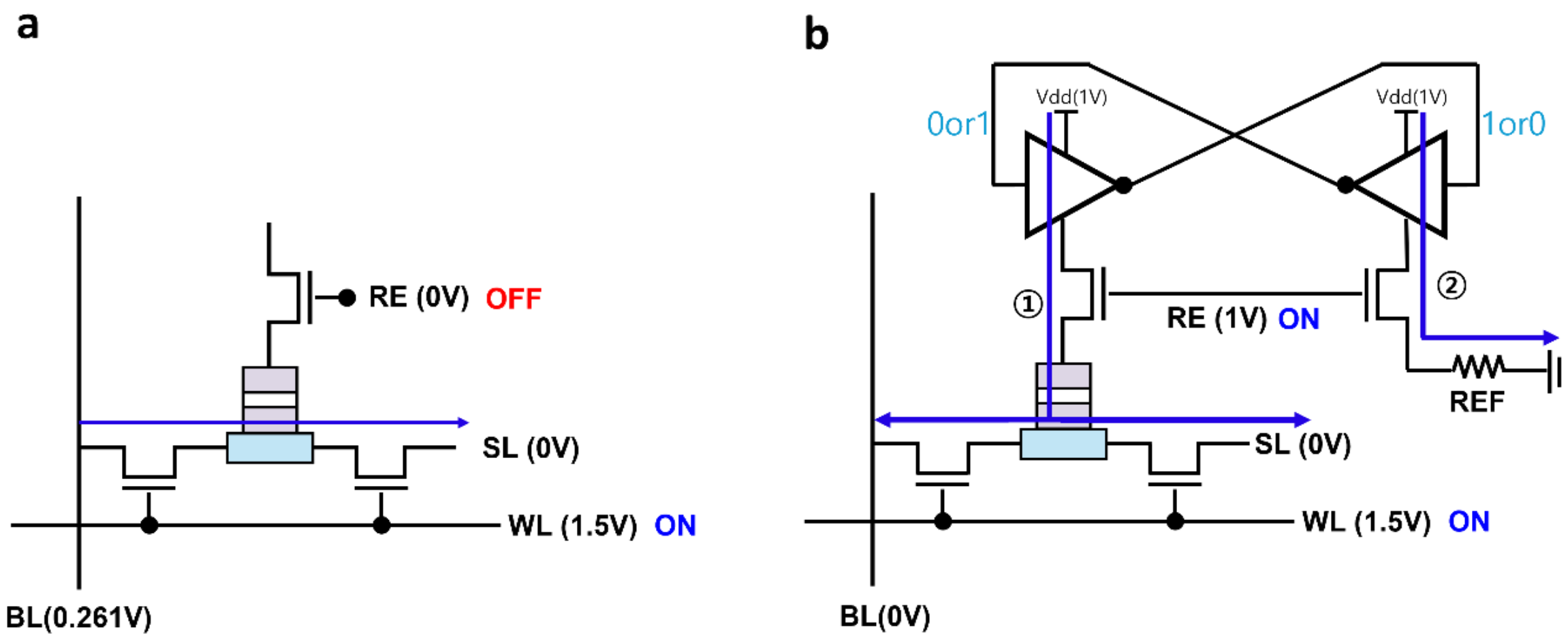

Figure S19. Schematic illustration of MRAM operations. a) Writing process. **b)** Reading process.

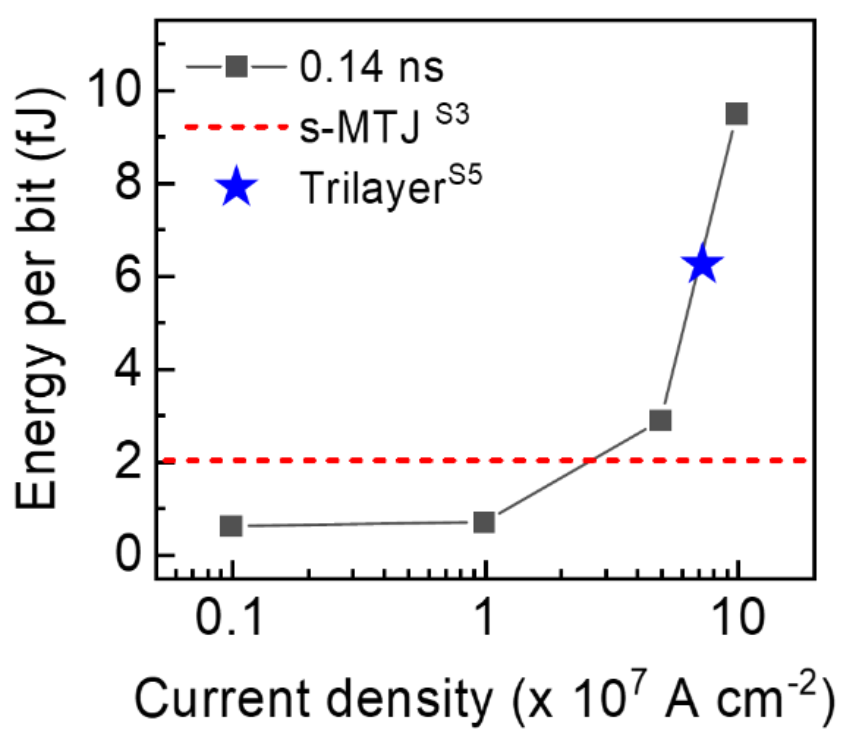

Figure S20. Energy consumption (per bit) calculation in the trilayer p-bits with different current density at a pulse width of 0.14 ns. Red dashed line indicates the energy consumption of s-MTJ[S4] Blue star indicates the energy consumption of the trilayer[S6].

13. Reproducibility and device-to-device variation

Figure S21 shows repeated measurements of the anomalous Hall effect (AHE) loop shift under different in-plane magnetic fields. The average values and corresponding standard deviations extracted from these measurements are used to generate the data and error bars shown in Fig. 3b of the main text.

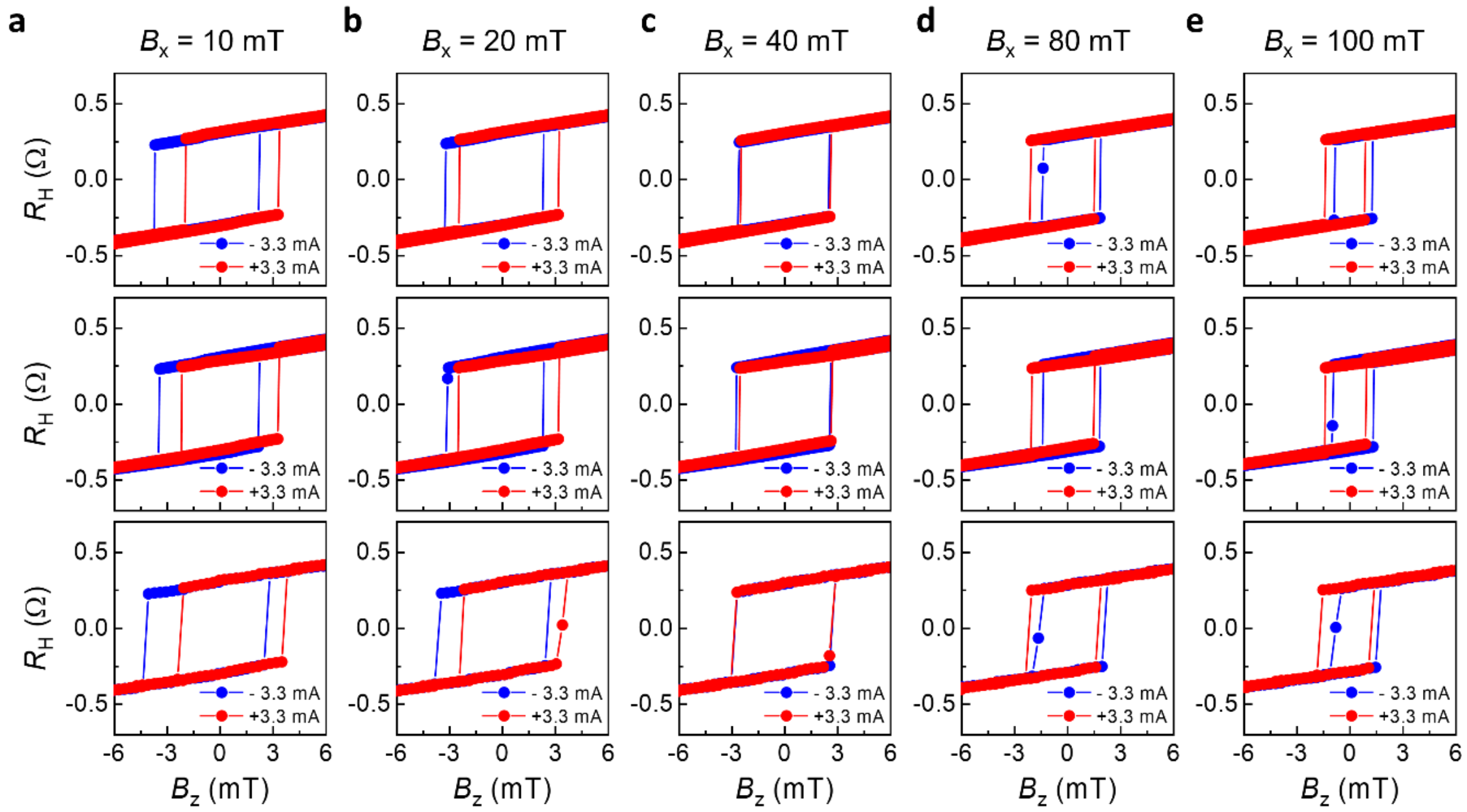

Figure S21. AHE loop shift in a Fe/Ti/CoFeB trilayer. a-e) AHE measurement results with d.c. current ±3.3 mA under different B_x of 10 mT (a), 20 mT (b), 40 mT (c), 80 mT (d), and 100 mT (e).

Figure S22 present the P_{UP} as a function of B_x while applying the same pulse current for three different devices. By fitting each dataset to a sigmoid function, we obtained a distribution of the center field (for P_{UP}=50%) is 7.15± 0.51 mT. The transition width, defined as the field range over which P_{UP} changes from 10 % to 90 %, is 5.05 ± 0.52 mT. The slope at the center, corresponding to the switching sensitivity, is 21.8 ± 2.2 %/mT. These variations are on the order of ~10%, indicating no significant device-to-device variation.

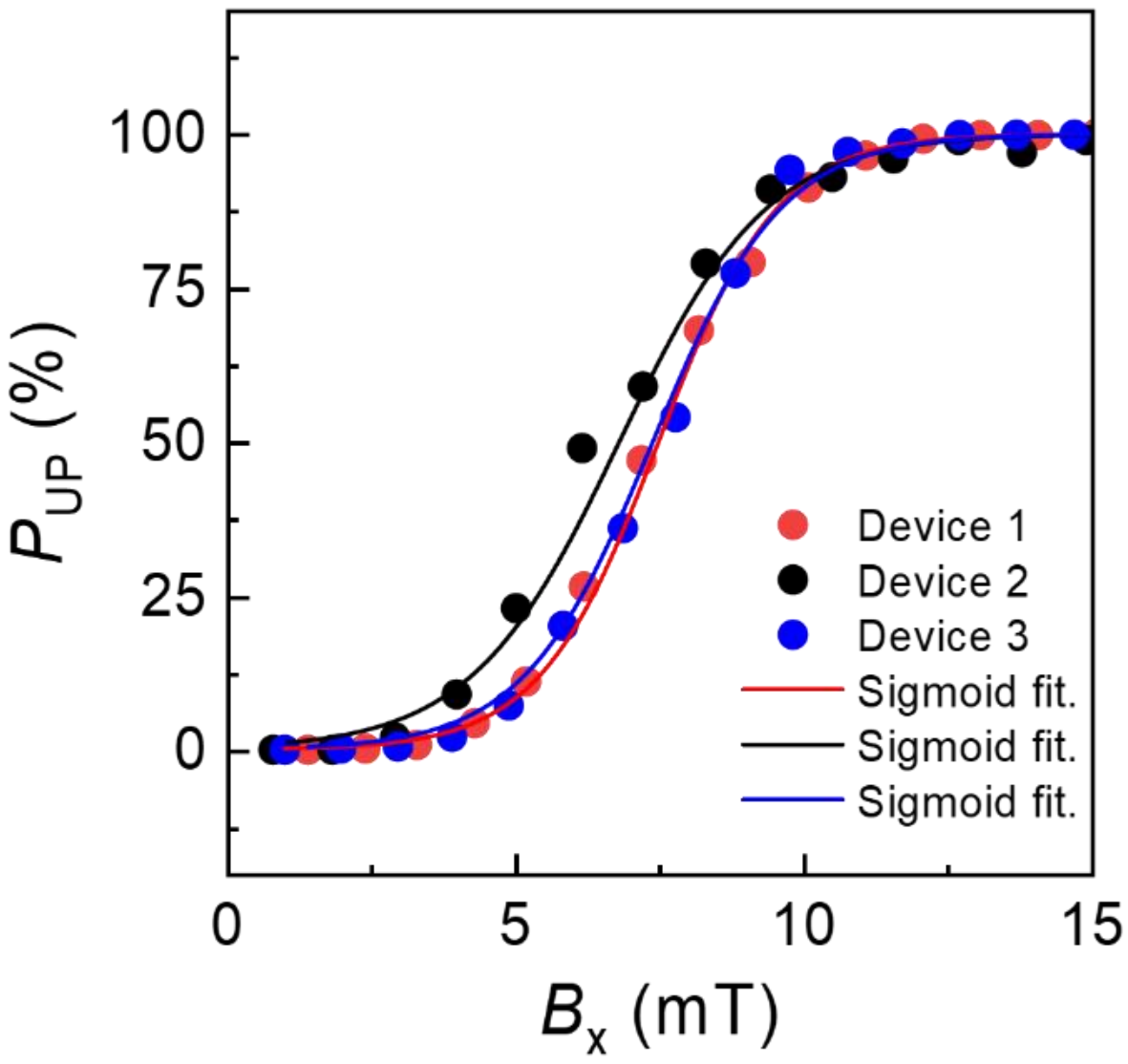

Figure S22| Device to device variation. P_{UP} as a function of B_x with I_a of 7 mA for three devices and sigmoidal fittings

14. Current-dependent AHE loop shift in an Fe/Ti/CoFeB trilayer

Figure S23 shows AHE loop shift measurements with different d.c. currents. As the current increases, ΔB_S is linearly increases (Figure S23d).

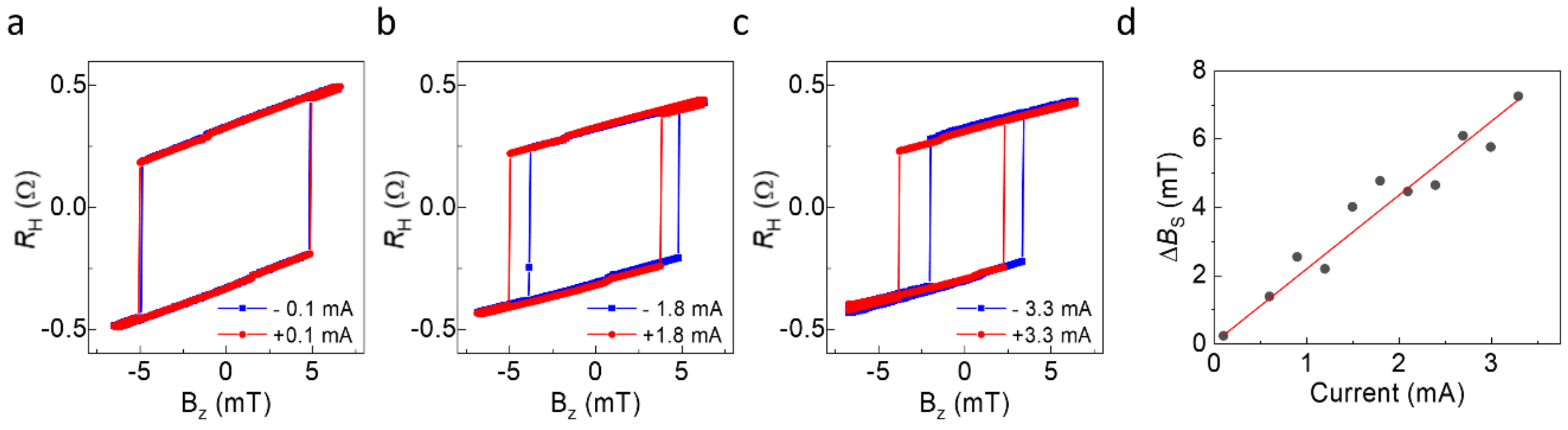

Figure S23. a-c) AHE loop shift measurements with different d.c. currents, ±0.1 mA (a), ±1.8 mA (b), ±3.3 mA (c). **d)** ΔB_S versus d.c. current

15. Derivation of the switching current density

We analyze the magnetization dynamics of $\hat{\mathbf{m}}$ using the Landau-Lifshitz-Gilbert (LLG) equation including the damping-like torque (DLT),

$$\partial_t\hat{\mathbf{m}} = -\gamma\hat{\mathbf{m}} \times \mathbf{B}_{eff} + \alpha\hat{\mathbf{m}} \times \partial_t\hat{\mathbf{m}} + \gamma h_D\hat{\mathbf{m}} \times (\hat{\mathbf{m}} \times \hat{\boldsymbol{\sigma}}), \quad \text{(S1)}$$

where γ is the gyromagnetic ratio, α is the Gilbert damping constant, and $\mathbf{B}_{eff}(= B_k(\hat{\mathbf{m}} \cdot \hat{\mathbf{z}})\hat{\mathbf{z}} + B_x\hat{\mathbf{x}})$ is the effective field consisting of the magnetic anisotropy (B_k) and the external field along the x-direction (B_x). The DLT coefficient is given by $h_D = \hbar\theta_{SH,D}J/2eM_st_F$, where $\theta_{SH,D}$ is the effective spin Hall angle, J is the current density, t_F is the thickness of the top ferromagnet layer, and $\hat{\boldsymbol{\sigma}}$ is a spin polarization vector defined as $\hat{\boldsymbol{\sigma}} = (0, \cos\eta, \sin\eta)$. The equations (2) and (3) of the manuscript describe the critical switching current densities for the two competing mechanisms in the ferromagnetic trilayer: the Anti-damping (STT-like) switching induced by the z-spin polarization ($\hat{\sigma}_z = \sin\eta$) and the Instability (SOT-like) switching induced by the y-spin polarization ($\hat{\sigma}_y = \cos\eta$).

The Anti-damping switching occurs via magnetization precession where the spin torque compensates the damping torque. For simplicity, since the switching is mainly driven by z-spin polarization, we consider only z-component of $\hat{\boldsymbol{\sigma}}$ in this switching condition. To solve for the precession amplitude, we transform the coordinate system from the laboratory frame (x, y, z) to a rotating frame (x', y', z') aligned with the equilibrium magnetization $\hat{\mathbf{m}}_{eq}$. This coordinate transformation is defined by the rotation angles $\theta = \tan^{-1}(B_x/B_k)$ (assuming $B_k \gg B_x$) and $\phi = 0$, which correspond to the magnetization tilt induced by the external field B_x.

The rotation matrix used in the derivation transforms the basis vectors. In this local frame, we assume a small amplitude precession ansatz for the magnetization $\hat{\mathbf{m}} = (a\cos 2\pi ft, a\sin 2\pi ft, 1)$, where a is the small precession amplitude and f is the resonance frequency. We substitute this ansatz into the LLG equation. The switching condition is met when the energy gain from the anti-damping torque equals the energy loss due to Gilbert

damping over one precession period. The z-component of time-integrated LLG equation results in,

$$Jh_D\gamma\cos\theta\sin\eta - 2\alpha\pi f = 0. \qquad \text{(S2)}$$

By solving the time-integrated equation for the current density J, the solution yields $J = J_{STT}$ with substituting $f = \gamma\sqrt{B_x^2 + B_k^2}/2\pi$,

$$J_{STT} = \pm\alpha\frac{2e}{\hbar}\frac{M_s t_F}{\theta_{SH,D}}B_k\left(1+\frac{B_x^2}{B_K^2}\right)\frac{1}{sin\,\eta}, \qquad \text{(S3)}$$

which corresponds to Eq. (2) of the manuscript. We note that the positive (negative) value of the J_{STT} indicates the switching condition where the magnetization flips from "UP" to "DOWN" ("DOWN" to "UP") when $sin\,\eta > 0$, and that the positive (negative) value of the J_{STT} indicates the switching condition where the magnetization flips from "DOWN" to "UP" ("UP" to "DOWN") when $sin\,\eta < 0$.

The instability switching mechanism in ferromagnetic trilayers is governed by the competition between the restoring torque arising from magnetic anisotropy and the destabilizing torques originating from the DLT and the external magnetic field [*Appl. Phys. Lett*. 102, 112410 (2013)]. For simplicity, since the switching is mainly driven by y-spin polarization, we consider only y-component of $\hat{\boldsymbol{\sigma}}$ in this switching condition. We consider the magnetization unit vector as $\hat{\mathbf{m}} = (\cos\phi\sin\theta, \sin\phi\sin\theta, \cos\theta)$. Denoting the right side of the Eq. (S1) as τ_{LLG}, the instability condition is met when the DLT and the external field overcome the anisotropy barrier, i.e., $\tau_{\text{LLG}} = 0$ and $\partial\tau_{\text{LLG}}/\partial\cos\theta = 0$. By solving these simultaneous equations analytically and applying a first-order approximation with respect to the external field B_x, we obtain

$$2h_D\cos\eta = B_k - \sqrt{2}B_x. \qquad \text{(S4)}$$

The equation (S4) can be realigned in terms of $J = J_{SOT}$,

$$J_{SOT} = \pm\frac{2e}{\hbar}\frac{M_s t_F}{\theta_{SH,D}}\left(\frac{B_k}{2}-\frac{|B_x|}{\sqrt{2}}\right)\frac{1}{cos\,\eta}, \qquad \text{(S5)}$$

which corresponds to Eq. (3) of the manuscript. We note that the positive (negative) value of the J_{SOT} indicates the switching condition where the magnetization flips from "UP" to "DOWN" or "DOWN" to "UP" directions. Flip conditions depend on the signs of B_x and $cos\,\eta$. For instance, the positive (negative) value of the J_{SOT} indicates the switching condition where the magnetization flips from "DOWN" to "UP" ("UP" to "DOWN") when $B_x > 0$ and $cos\,\eta < 0$.

Eq. (4) of the manuscript defines the boundary condition where the switching probability is 50%, separating the STT-dominant and SOT-dominant switching regimes. Considering the magnetization unit vector as $\hat{\mathbf{m}} = (\cos\phi\sin\theta\,, \sin\phi\sin\theta\,, \cos\theta)$, the boundary condition is derived by solving the LLG equation for a static solution ($\partial_{\mathrm{t}}\hat{\mathbf{m}} = 0$) under the condition that the magnetization lies within the plane ($\theta = \pi/2$). From the solution, we obtain the condition in terms of $J = J_{STT-SOT}$ with a stable equilibrium at $\phi = \pm\pi/2$ (along the y-axis),

$$J_{STT-SOT} = \pm\frac{2e}{\hbar}\frac{M_S t_F}{\theta_{SH,D}}\frac{B_x}{\sin\eta}. \qquad \text{(S6)}$$

which corresponds to Eq. (4) of the manuscript.

16. LLG simulations of magnetization switching in a magnetic trilayer

Figures S24a,b show P_{UP} as functions of B_x and J for different initial magnetization states, 'UP' and 'DOWN' magnetization states, respectively. Note that both *z*-SOT and *y*-SOT switching dominant regions are independent of the initial magnetization state.

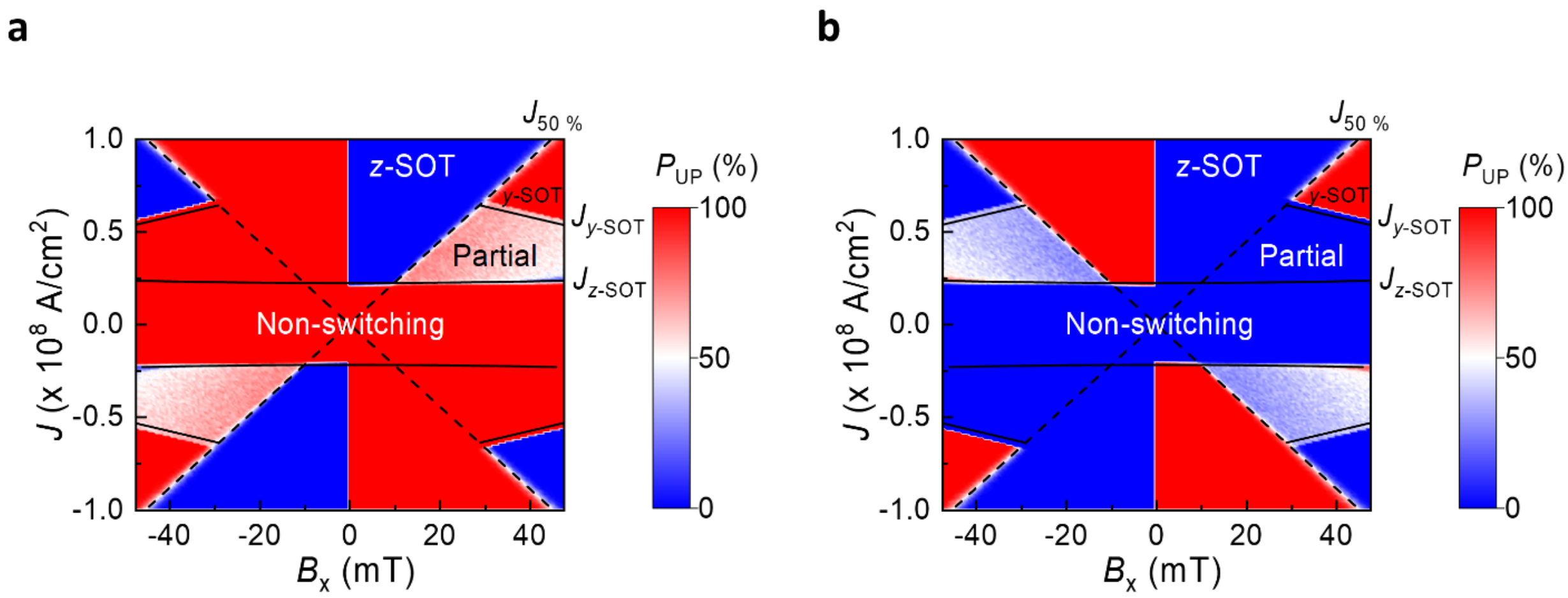

Figure S24. a,b) P_{UP} of the magnetic trilayer as functions of B_x and J for initial magnetization of 'UP' (a) and 'DOWN' (b) directions.

17. Forward AND gate operations

We performed forward 'AND' logic operations with the same circuit used in Figure 5 of the main text. Here, we set the input x_1, x_2 as (0,0) (1,0), (0,1), and (1,0) and obtained the probability of the output x_3, P(x_3). Figure S25 shows the results that for all input conditions, the probability of a correct answer is greater than 85%.

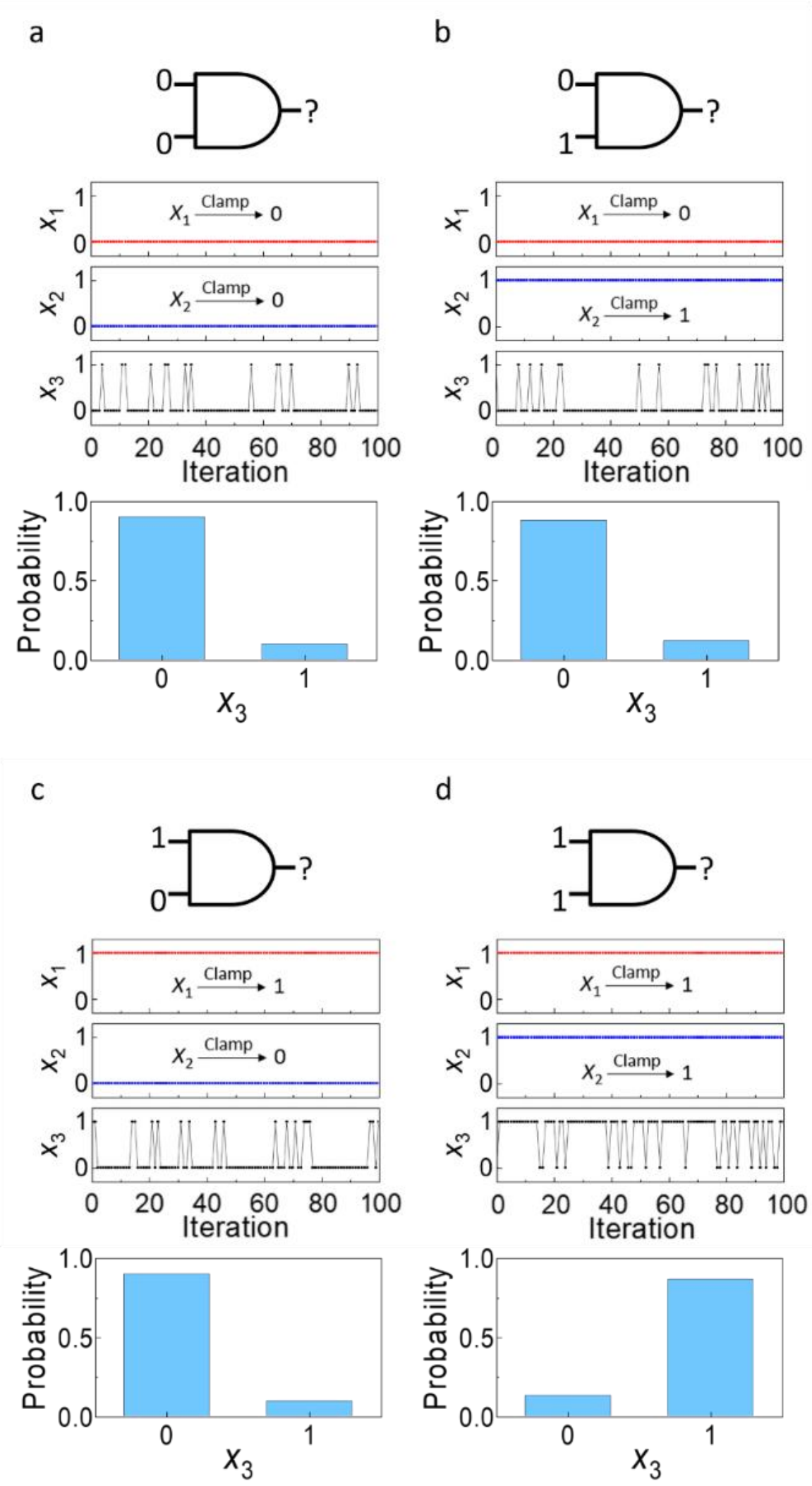

Figure S25. a-d) The results of the forward 'AND' gate operation. Top panel: p-bit streams of three p-bit devices (x_1, x_2, x_3) for 100 iterations. Bottom panel: the probabilities of outputs (x_3) obtained from when input (x_1, x_2) is set to (0,0) (a), (0,1) (b), (1,0) (c), and (1,1) (d).

18. P-bit streams with 16 different probabilities

Figure S26 shows generated p-bit streams with different P_{UP} from 0/16, 1/16, … , 14/16, and 15/16.

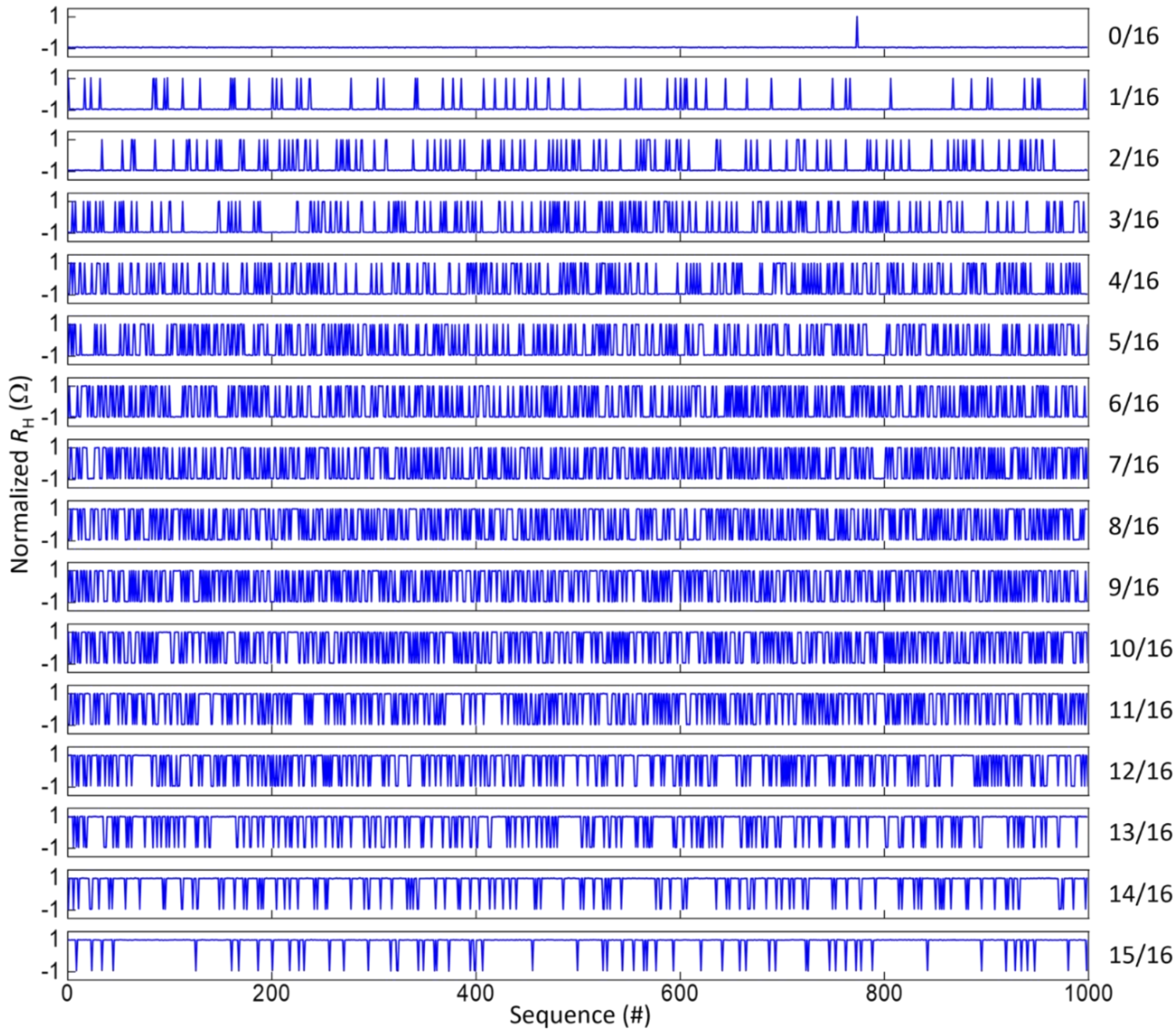

Figure S26. P-bit streams with different probabilities P_{UP}.

19. MAC operations of p-bit streams

Figure S27 shows the MAC operation results of p-bit streams used in Figure 5 of the main text.

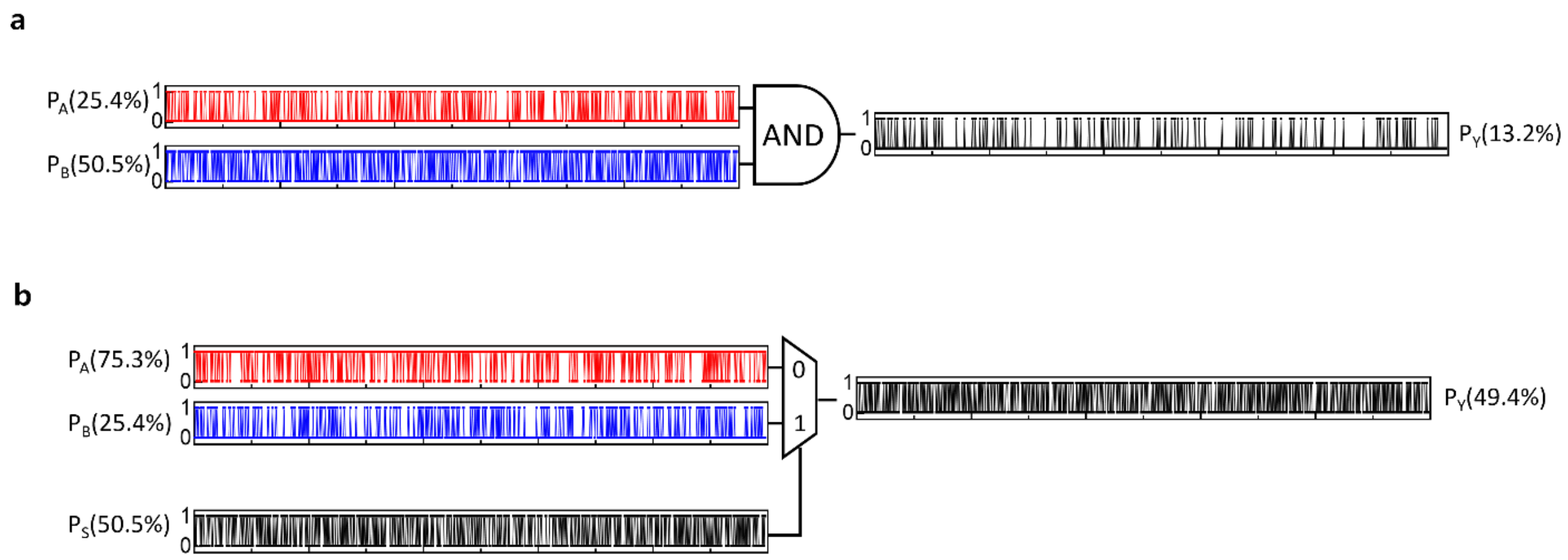

Figure S27. MAC operation results of p-bit streams. a) Multiplication by AND gate. **b)** Addition by MUX circuit.

20. Comparison of energy and area consumption of stochastic neural networks

We compared the performance of conventional and p-computing hardwares using an N-bit 10-input MAC operation. Figure S28a illustrates a conventional binary deterministic computing hardware, containing 10 multipliers with N-bit inputs and an adder tree that sums the results for the final MAC output. In contrast, Figure S28b shows a p-computing hardware based on the trilayer p-bits demonstrated in the main text. Initially, the trilayer device generates p-bit streams representing the probabilities of the N-bit input. Each cycle, 10 'AND' gates operate on the bit stream, and their results are accumulated by a parallel counter. Note that the conventional computing hardware was implemented following common practice[S12,S13], while the p-computing hardware was implemented based on refs. [S14 and S15]. For the performance evaluation, the p-bit-based circuits were simulated using Virtuoso. The MAC units were implemented through Verilog RTL design and synthesized using Synopsys Design Compiler with a 28nm CMOS standard cell library. Both MAC units operate at 1 GHz, and their power consumption was simulated using Synopsys PrimeTime PX. Figure S28c shows the performance comparison between conventional deterministic computing and p-computing. For 4-bit operation, trilayer based p-computing achieves a 39% reduction in energy-area product (EAP) compared to conventional computing. Notably, the reduction in EAP becomes even more significant as the bit-width increases. This is because p-computing maintains consistent area usage and improves power efficiency regardless of bit precision, whereas area and power consumption scale with bit-width for conventional computing. We attribute the area and power efficiency of the p-computing to the simplicity of 'AND' gates compared to multipliers for conventional computing.

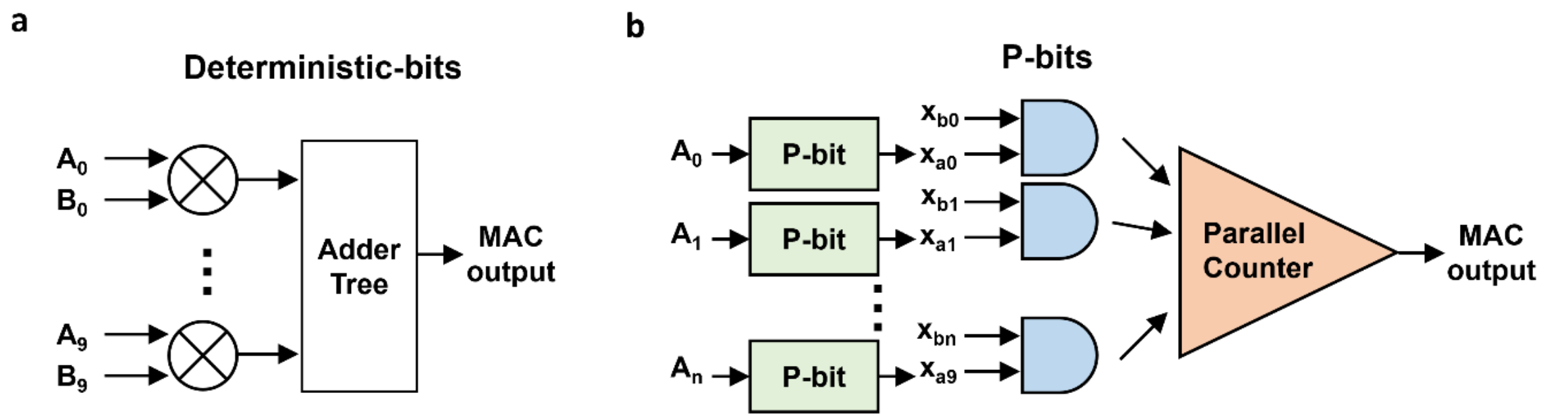

c

	Deterministic computing					P-bit based computing
MAC input	4-bit	5-bit	6-bit	7-bit	8-bit	N-bit
Area (μm^2)	2032	3189	4893	5394	6390	1079 (189.6)
Energy (nJ)	1.13	1.59	2.39	2.63	3.15	1.29 (0.0209)
EAP ($\mu m^2 * \mu J$)	2.296	5.070	11.694	14.186	20.129	1.39
Reduction rate (%)	39.5	72.6	88.1	90.2	93.1	-

Figure S28. a) Schematic of conventional deterministic computing hardware for MAC operation. **b)** Schematic of p-computing hardware for MAC operation. **c)** Performance comparison between conventional computing and p-computing for n-bit 10-input MAC operation. The value in parentheses corresponds to the trilayer p-bit stream generator, where the bit-stream length was set to 100.

21. Integer factorization and Max-Cut problems based on trilayer p-bits

We conducted two additional computing tasks of a different category: 6-bit integer factorization and Max-Cut problem. Integer factorization is a combinatorial problem that requires searching a large discrete solution space and the Max-Cut problem is a representative NP-hard problem that seeks to partition a graph into two sets to maximize the sum of the weights of the edges between them. Both problems demand exploration of significantly larger state spaces and longer effective bit streams compared to the previous functional tasks. We implemented both tasks using a unified probabilistic update rule[S16]

$$m_i(t) = \mathrm{sgn}[\mathrm{rand}(-1,+1) + \tanh(I_i(t))], \quad m_i \in \{-1,+1\},$$

where $m_i(t)$ denotes the binary state (+1 or −1) of the i-th p-bit at time t. The term $\mathrm{rand}(-1,+1)$ is a uniform random variable, which is used by the experimentally measured random bits in our study. The activation function $\tanh(I_i)$ reflects the sigmoid-like behavior obtained from experimental characterization in Figure 2d in main text. The input $I_i(t)$ is computed as:

$$I_i(t) = \sum_j J_{ij}\, m_j(t) + h_i,$$

where J_{ij} encodes the coupling strength between p-bits i and j, and h_i is an optional bias term. In the 6-bit factorization task, the output p-bits representing the product Z were clamped (e.g., to 001111 for $Z = 15$), while the inputs X, Y, and internal logic nodes were allowed to fluctuate. Over 8000 stochastic updates, the system successfully identified the correct factor pairs (e.g., (5, 7) for $Z = 35$) with the highest frequency, as shown in Figure S29. This demonstrates that a 21-p-bit network can solve inverse arithmetic problems through hardware-based probabilistic inference.

For the Max-Cut problem, we used instances from the Biq Mac Library[S17], which is a standard benchmark for optimization algorithms. In this formulation, each p-bit represents a graph node, and its binary state indicates its partition assignment. The coupling term J_{ij}

corresponds to the edge weight between nodes i and j. In the case of the 800-node Max-Cut instance, we performed 100 independent runs, each with 1000 steps. As shown in Figure S30a, the system rapidly converged: the average Max-Cut value reached 11578.21 within just 200 annealing steps, very close to the best-known value of 11624. Similarly, for a 2000-node Max-Cut problem from the same library, the average value converged to 13288.61 within 200 steps, compared to the best-known value of 13359 (Figure S30b). These results confirm that our approach not only scales well with problem size but also achieves near-optimal performance efficiently, without requiring prolonged annealing

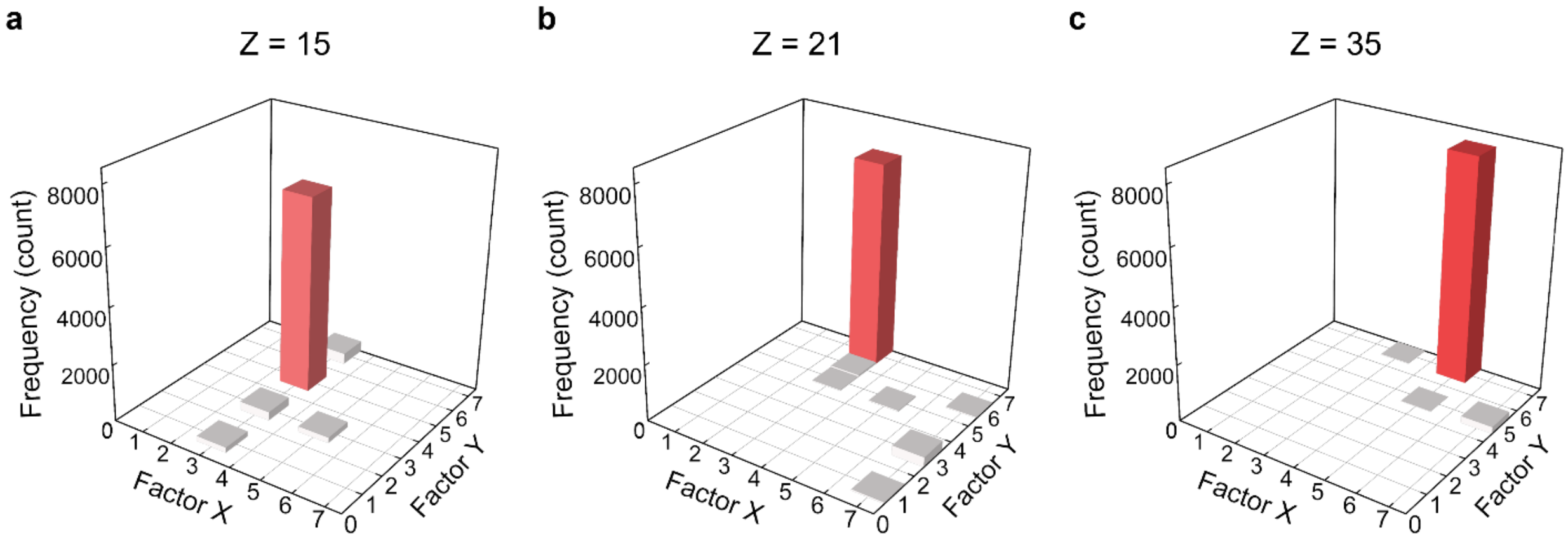

Figure S29. P-bit based factorization results. a) Z=15. **b)** Z=21, **c)** Z=35.

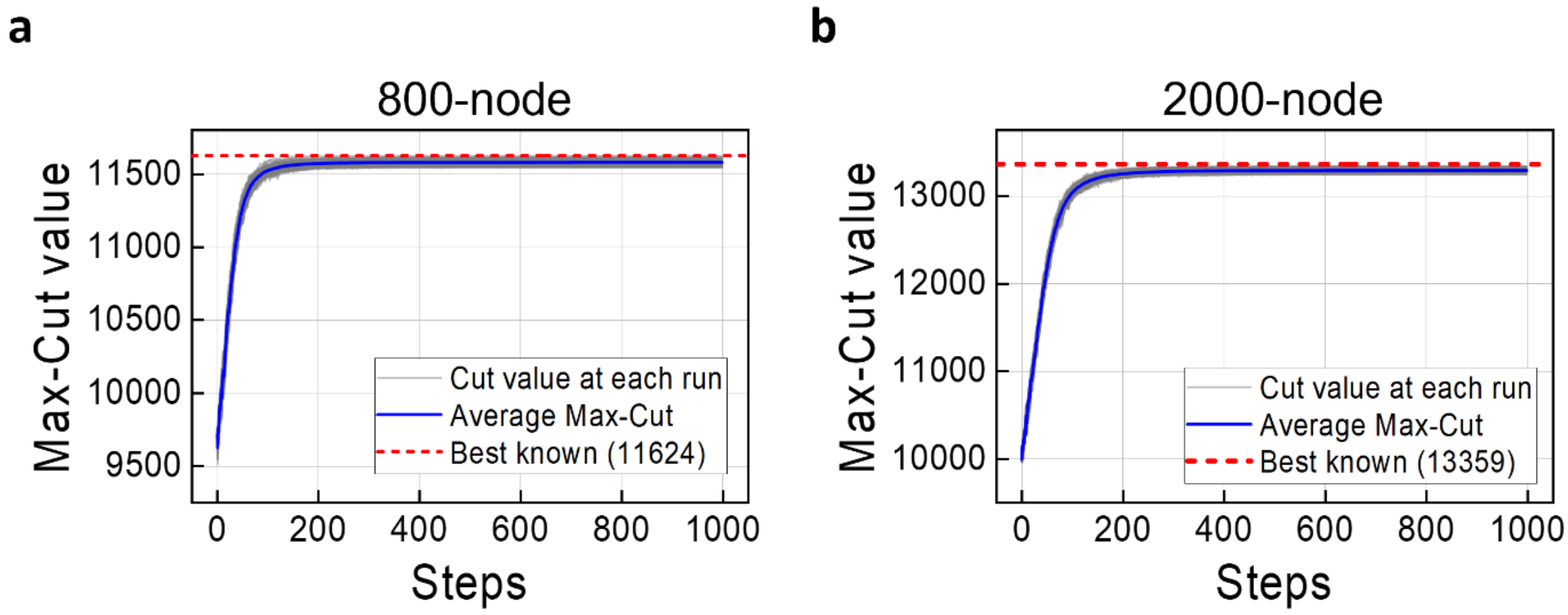

Figure S30. P-bit based max-cut problems results. a) 800-node problem. **b)** 2000-node problem

References

[S1] Rukhin, A., et al., "A Statistical Test Suite for Random and Pseudorandom Number Generators for Cryptographic Applications," *NIST Special Publication* **2010**, 800–822.

[S2] Kim, T., et al., "Demonstration of in-plane magnetized stochastic magnetic tunnel junction for binary stochastic neuron," *AIP Advances* **2022**, 12, 075104. https://doi.org/10.1063/5.0090577

[S3] Yang, K., et al., "16.3 a 23mb/s 23pj/b fully synthesized true-random-number generator in 28nm and 65nm CMOS," in *Proceedings of the International Solid-State Circuits Conference (ISSCC)*, IEEE, **2014**, 280–281. https://doi.org/10.1109/ISSCC.2014.6757434

[S4] Shan, W., Fan, A., Xu, J., Yang, J., Seok, M., "A 923 gbps/w, 113-cycle, 2-SBOX energy-efficient AES accelerator in 28nm CMOS," in *2019 Symposium on VLSI Circuits*, IEEE, **2019**, C236–C237. https://doi.org/10.23919/VLSIC.2019.8778189

[S5] Ahmed, I., et al., "A comparative study between spin-transfer-torque and spin-Hall-effect swtiching mechanisms in PMTJ using SPICE," IEEE JXCDC, **2017**, 3, 74-82. https://doi.org/10.1109/JXCDC.2017.2762699

[S6] Borders, W. A., et al., "Integer factorization using stochastic magnetic tunnel junctions," *Nature* **2019**, 573, 390–393. https://doi.org/10.1038/s41586-019-1557-9

[S7] Hayakawa, K., et al., "Nanosecond random telegraph noise in in-plane magnetic tunnel junctions," *Physical Review Letters* **2021**, 126, 117202. https://doi.org/10.1103/PhysRevLett.126.117202

[S8] Yang, Q., et al., "Field-free spin–orbit torque switching in ferromagnetic trilayers at sub-ns timescales," *Nature Communications* **2024**, 15, 1814. https://doi.org/10.1038/s41467-024-46113-1

[S9] Grimaldi, E., et al., "Single-shot dynamics of spin–orbit torque and spin transfer torque switching in three-terminal magnetic tunnel junctions," *Nature Nanotechnology* **2020**, 15, 111–117. https://doi.org/10.1038/s41565-019-0607-7

[S10] Krizakova, V., Garello, K., Grimaldi, E., Kar, G. S., Gambardella, P., "Field-free switching of magnetic tunnel junctions driven by spin–orbit torques at sub-ns timescales," *Applied Physics Letters* **2020**, 116, 232406. https://doi.org/10.1063/5.0011433

[S11] Polley, D., et al., "Picosecond spin–orbit torque–induced coherent magnetization switching in a ferromagnet," *Science Advances* **2023**, 9, eadh5562. https://doi.org/10.1126/sciadv.adh5562

[S12] Chen, Y.-H., Krishna, T., Emer, J. S., Sze, V., "Eyeriss: an energy-efficient reconfigurable accelerator for deep convolutional neural networks," *IEEE Journal of Solid-State Circuits* **2016**, 52, 127–138. https://doi.org/10.1109/ISSCC.2016.7418007

[S13] Chen, T., et al., "DianNao: a small-footprint high-throughput accelerator for ubiquitous machine-learning," *ACM SIGPLAN Notices* **2014**, 49, 269–284. https://doi.org/10.1145/2644865.2541967

[S14] Sim, H., Lee, J., "A new stochastic computing multiplier with application to deep convolutional neural networks," in *2017 54th ACM/EDAC/IEEE Design Automation Conference (DAC)*, IEEE, **2017**, 1–6. https://doi.org/10.1145/3061639.3062290

[S15] Yu, J., Kim, K., Lee, J., Choi, K., "Accurate and efficient stochastic computing hardware for convolutional neural networks," in *2017 IEEE International Conference on Computer Design (ICCD)*, IEEE, **2017**, 105–112. https://doi.org/10.1109/ICCD.2017.24

[S16] Camsari, K. Y., Faria, R., Sutton, B. M., Datta, S., "Stochastic p-bits for invertible logic," *Physical Review X* **2017**, 7, 031014. https://doi.org/10.1103/PhysRevX.7.031014

[S17] Wiegele, A., "Biq Mac Library – a collection of Max-Cut and quadratic 0–1 programming instances of medium size," *University of Klagenfurt Technical Report* **2007**. https://biqmac.aau.at/biqmaclib.pdf
